# Microbial bile salt hydrolase activity influences gene expression profiles and gastrointestinal maturation in infant mice

**DOI:** 10.1080/19490976.2022.2149023

**Published:** 2022-11-24

**Authors:** María A. Núñez-Sánchez, Florence M. Herisson, Jonathan M. Keane, Natalia García-González, Valerio Rossini, Jorge Pinhiero, Jack Daly, Milán Bustamante-Garrido, Cara M. Hueston, Shriram Patel, Nuria Canela, Pol Herrero, Marcus J. Claesson, Silvia Melgar, Ken Nally, Noel M. Caplice, Cormac G.M. Gahan

**Affiliations:** a APC Microbiome Ireland, University College Cork, Cork, Ireland; b Obesity and Metabolism Laboratory, Biomedical Research Institute of Murcia (IMIB-Arrixaca), Murcia, Spain; c Centre for Research in Vascular Biology, University College Cork, Cork, Ireland; d School of Microbiology, University College Cork, Cork, Ireland; e Eurecat, Centre Tecnològic de Catalunya, Centre for Omic Sciences (COS), Joint Unit Universitat Rovira I Virgili-EURECAT, Unique Scientific and Technical Infrastructures (ICTS), Reus, Spain; f School of Biochemistry & Cell Biology, University College Cork, Cork, Ireland; g School of Pharmacy, University College Cork, Cork, Ireland

**Keywords:** Bile, organoid, colon, neonatal, infant, gut development, intestine, stem cell

## Abstract

The mechanisms by which early microbial colonizers of the neonate influence gut development are poorly understood. Bacterial bile salt hydrolase (BSH) acts as a putative colonization factor that influences bile acid signatures and microbe-host signaling pathways and we considered whether this activity can influence infant gut development. *In silico* analysis of the human neonatal gut metagenome confirmed that BSH enzyme sequences are present as early as one day postpartum. Gastrointestinal delivery of cloned BSH to immature gnotobiotic mice accelerated shortening of the colon and regularized gene expression profiles, with monocolonised mice more closely resembling conventionally raised animals. *In situ* expression of BSH decreased markers of cell proliferation (Ki67, Hes2 and Ascl2) and strongly increased expression of ALPI, a marker of cell differentiation and barrier function. These data suggest an evolutionary paradigm whereby microbial BSH activity potentially influences bacterial colonization and in-turn benefits host gastrointestinal maturation.

## Introduction

The establishment of a stable gut microbiota drives the functional development of the gastrointestinal tract (GIT), though the microbial factors that influence gut development are currently unclear. The classical pattern of gut microbial colonization of the GIT in healthy newborns involves early establishment of facultative anaerobes (predominantly members of the family Enterobacteriaceae) but this gradually changes during the first week of life toward a microbiota rich in *Bifidobacterium* ssp.,^1^ along with other bacteria including *Enterococcus* and *Lactobacillus*.^2^ The increase in microbial diversity is coincident with both the development of the immune system and rapid morphological and functional gut maturation. These changes in early life also affect the production of microbial-derived metabolites, many of which have the potential to influence host signaling processes.^3,4^

In particular, bile acids (BA) have the capacity to influence cellular processes in the host. BA are synthesized in hepatocytes from cholesterol and released into the intestine conjugated to either taurine or glycine. Bacterial bile salt hydrolase (BSH) enzymes in the gut significantly modify BA by deconjugation to generate unconjugated BA, which are then subject to further bacterial modifications to yield secondary and tertiary BA.^5^ BSH activity is widely distributed across the major bacterial divisions in the GIT including *Lactobacillus, Bifidobacterium, Enterococcus, Clostridium* and *Bacteroides* spp as well as the gut Archea.^6,7^ Previous studies from our lab demonstrated that cloning and expression of BSH in a Gram positive bacterium normally lacking this activity (*Listeria innocua*) significantly improved microbial bile tolerance *in vitro* and enhanced gut colonization in mice.^7^ This functional activity and evidence for host-driven selection of this trait in gut bacteria suggests that BSH may represent an evolutionary microbial adaption promoting stable gut colonization.^7,8^ Further studies from our group showed that gut-localized BSH activity significantly impacts host gene expression profiles reflecting physiological processes (including regulation of lipid metabolism, local immune function and gut development and homeostasis) in adult germ-free (GF) and conventionally raised (Conv) mice.^9^ The work supports other studies which suggest a significant role for bacterial BA metabolism in regulating host metabolic and immune functions^10,11^ as well as a role for BAs as modulators of microbial community structure in the infant gut.^12^ As we previously associated BSH activity with induction of gene systems involved in epithelial cell differentiation and homeostasis,^9^ the current study was designed to determine whether BSH activity and subsequent BA signatures may play a role in the development and maturation of the gut in infant mice.

Gastrointestinal monocolonization of 3-week-old gnotobiotic mice with engineered *E. coli* heterologously expressing potent BSH activity reduced markers of mucosal cell proliferation and increased expression of intestinal alkaline phosphatase (ALPI), a marker of enterocyte differentiation. BSH also influenced gut development (in a similar manner to Conv animals) in contrast to the delayed development of the gut in GF animals or mice monocolonised by *bsh^−^* wild-type *E. coli*. In support of these findings exposure of a colon organoid model to a mixture of unconjugated bile acids and lipopolysaccharide (LPS) promoted an increase in organoid size, a decrease in organoid number and a decrease in LGR5 (a marker of stemness) indicating a role for BA in the regulation of stem cell proliferation.

## Results

### BSH-encoding genes can be detected in the human infant gut microbiome as early as one day postpartum

Microbial BSH activity is assumed to be present in the early neonatal gut but to our knowledge quantitative evidence is lacking. Here, we provide direct evidence that genes encoding BSH activity are present as early as one-day postpartum in the human infant microbiome (Supplementary material 1). Analysis of published shotgun metagenomic data in a controlled study of infants between 1 day and 4 months postpartum^13^ demonstrated the presence of *bsh* genes of potential *Bacteroides, Lactobacillus*, and *Bifidobacterium* origin. We appreciate that the approach may fail to detect *bsh* genes from other genera and further studies will be necessary to determine functional BSH activity. However, the analysis demonstrates the potential for this enzymatic activity to occur in neonates and supports and extends a recent study which demonstrates BSH activity in infants at 1 month of age but did not examine earlier timepoints.^14^ In our analysis, we investigated metagenomes from the tongue dorsum in the same study^13^ as a control and failed to identify *bsh* genes in this environment (Supplementary material 1), illustrating the gut-adapted nature of BSH activity.^7^

### Expression of BSH in *Escherichia coli* MG1655 and colonization in germ free mice

**Figure 1.**
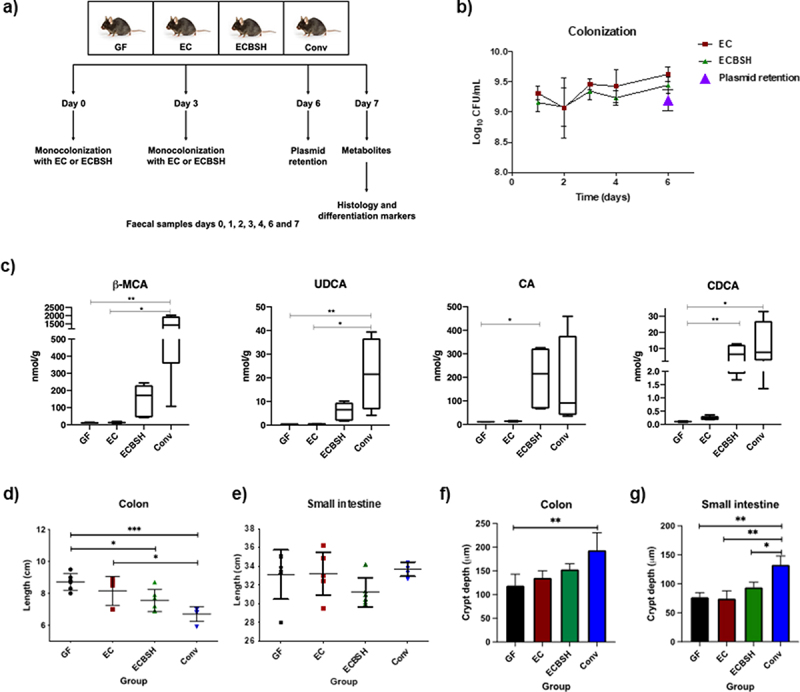
BSH activity and effect on colon length. a) Study overview. Female C57BL/6 GF mice (n = 6) (aged 3–4 weeks) were monocolonized with 10^9^ EC or ECBSH. Control groups of age-matched GF mice and Conv C57BL/6 mice were included. Sampling points for colonization during the experiment and final sampling collection are indicated; b) *E. coli* colonization and plasmid retention capacity. Fecal samples were plated during the experiment to determine the colonization capacity. *In vivo* plasmid retention was evaluated at day 6 post-colonization; c) Bacterial BSH activity increases the production of primary unconjugated bile acids in infant mice; d) Bacterial BSH reduces colon length after 7 days of colonization. Colon length differences between groups were determined at the end of the experiment; e) Differences in small intestine length after 7 days of colonization; f) Crypt depth differences in colon samples. Values are given in μm; g) Crypt depth differences between groups in small intestine samples. Values are given in μm; P values were calculated using ANOVA test in those parameters with a normal distribution (Colonization, colon length, small intestine length, and crypt depth), whereas P values for parameters without a normal distribution (β-MCA, UDCA, CA, and CDCA) were calculated using a Kruskal-Wallis test (considering P < .05 significant), followed by a Tukey or Dunn’s post hoc analysis for the intergroup differences test respectively in those parameters with a significant P value. All values are represented as mean ± standard deviation. * p < .05; ** p < .01; *** p < .001. β-MCA, beta muricholic acid; UDCA, ursodeoxycholic acid; CA, cholic acid; CDCA, chenodeoxycholic acid.

To examine the potential effects of bacterial BSH activity in gut development in the early stages of life, 3 week old GF mice were monocolonized with a nonpathogenic *E. coli* MG1655 strain expressing a *bsh* gene from *Lactobacillus salivarus* JCM1046, which has been previously shown to affect host metabolism and homeostasis.*^9^* We utilized a multicopy pEX plasmid to express the *L. salivarius* JCM1046 *bsh1* allele (accession number FJ591081.1) under a P44 promoter for elevated expression (Supplementary material 2a). The functional activity of the engineered *E. coli* was confirmed by performing a taurodeoxycholic acid (TDCA) agar plate assay (Supplementary material 2b). GF mice were gavaged with *bsh^−^* wild-type *E. coli* (EC) or *E. coli* expressing BSH (ECBSH) as outlined, with GF and Conv serving as controls (Figure 1a). We determined that both *E. coli* expressing BSH (ECBSH group) or the *bsh^−^* wild-type *E. coli* (EC group) were capable of stably mono-colonizing the gut to high levels in 3 week old GF mice with good plasmid stability for the duration of the experiment (Figure 1b).

To confirm that ECBSH colonization functionally influenced *in vivo* BA signatures the BA profile was determined in feces by UHPLC-(ESI)-MS/MS. A total of 22 BA were analyzed in the feces and full results are shown in Supplementary material 3a. We recognize that not all BAs are represented in our analysis however we focused upon the most abundant murine BAs in order to demonstrate *in vivo* efficacy of the ECBSH strain in generating localized unconjugated bile acid signatures in the gut (feces). The unconjugated BAs cholic acid (CA) and chenodeoxycholic acid (CDCA) were significantly elevated by ECBSH (but not by EC) in the feces relative to GF mice (Figure 1c). Interestingly, TUDCA was detectable in GF mice as has been reported previously^15^ and we could detect unconjugated UDCA in the feces of mice colonized by ECBSH (Figure 1c). Overall levels of BA excretion in GF mice were lower than CONV animals as reported previously^15^ though the differences were not statistically significant. We determined an increase in the expression of genes encoding organic solute transporter α (Ost α) and organic solute transporter β (Ostβ) in the ileum of infant mice colonized by ECBSH (but not EC) in line with expression levels in Conv mice suggesting normalization of bile acid transport, a factor that could influence molecular signaling in our system (Supplementary material 4). Analysis of gene expression in the livers of mice demonstrated reduced expression of Cyp7a in Conv mice relative to GF mice as described previously,^11^ but no alteration in expression in monocolonised infant animals.

Previous studies have suggested a role for short-chain fatty acids in gut homeostasis and stem cell proliferation in the GIT.^16,17^ We predicted that microbial SCFA should not be produced in our model system and confirmed this by demonstrating that the SCFA profile did not change in the GF, EC, or ECBSH groups, although as expected it was different in Conv mice (Supplementary material 3b).

### Bacterial BSH activity impacts colon development in infant GF mice

Seven days after the first administration of bacteria, mice were sacrificed and evaluated for structural changes in the small intestine (SI), colon (COL) and cecum. SI, COL lengths and cecum weights were compared between monocolonized gnotobiotic mice and controls (untreated GF mice and Conv mice). Colonization with ECBSH, but not EC, induced a significant decrease in COL length when compared to the GF group (Figure 1d) indicative of an influence of BSH activity upon development of the colon in these animals. No alterations to SI length were determined in these groups (Figure 1e). In order to evaluate structural changes in the murine gut, we analyzed crypt structure and effects on the mucus layer using the Alcian blue/PAS assay (representative images for the COL are shown in Supplementary material 5a/b). Whilst we did not see full differentiation of the mucosa, we determined a trend toward increases in crypt depth, mucous depth and goblet cell number in ECBSH mice (Figure 1f**/g** and Supplementary material 5c/d). We suggest that additional microbial factors (such as SCFA) may be required for full differentiation of the gut in this model.^17^ Indeed, recent work has demonstrated that BA and SCFA signals may combine to regulate cellular processes in the gut.^18^

### BSH activity promotes altered transcriptional profiles in colonic epithelial cells in infant mice

**Figure 2.**
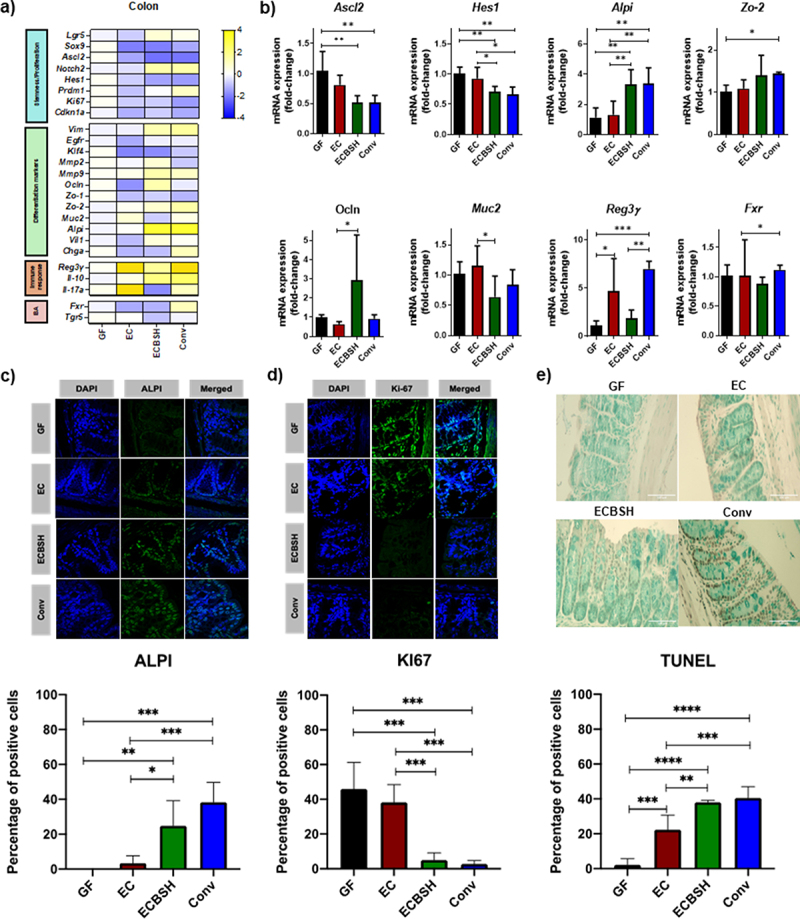
Bacterial BSH influences the host transcriptional landscape in infant mice. a) Colon murine gene expression profile in response to gastrointestinal colonization by qRT-PCR. Represented as the fold change between the condition and the GF group; b) Significant changes in stemness maintenance and differentiation genes by RT-qPCR. All values are represented as the fold change of relative mRNA expression between the condition and the GF group; c) Representative images of ALPI expression by confocal microscopy. Values are represented as the difference in percentage ALPI positive cells divided by the total of cells determined by DAPI between the condition and the GF group in at least 15 different fields for each animal. d) Representative images of Ki-67 expression by confocal microscopy. Values are represented as the difference in percentage Ki-67 positive cells divided by the total of cells determined by DAPI between the condition and the GF group in at least 15 different fields for each animal; e) Representative images of apoptotic cells determined by the TUNEL assay. Values are represented as the difference in percentage positive cells divided by the total of cells between the condition and the GF group in at least 15 different fields for each animal. For all microscopy analyses Colon samples from 6 mice per group were prepared, stained and 10 fragments from each tissue were further analyzed. Statistical analyses were conducted using one-way ANOVA and Tukey’s multiple comparison test for those parameters with normal distribution (*Hes1, Alpi, Zo-2, Ocln, Reg3γ, Fxr*, ALPI and Tunel assay), whereas Kruskal-Wallis test followed by a Dunn’s post hoc analysis was used with those parameters without normal distribution (*Ascl2, Muc2* and KI67). All values are represented as mean ± standard deviation. * p < .05; ** p < .01; *** p < .001; **** p < .00001.

Given the observed change in COL length, we used RT-qPCR to analyze a total of 30 genes involved in WNT and Notch signaling pathways, and differentiation-related genes, including markers of enterocytes, Goblet cells and enteroendocrine cells, and BA receptors (Figure 2a, Supplementary material 6). Figure 2b presents graphical representation of data for all genes in which there were statistically significant changes between mouse groups. The results showed that BSH activity promotes a significant decrease in the expression of the transcription factor achaete scute-like 2 (Ascl2), a regulator of stem cell identity and the transcription repressor hairy and enhancer of split 1 (Hes1), a marker of absorptive progenitor cells. Of note, we also observed that BSH activity promoted a significant increase in the expression of intestinal alkaline phosphatase (Alpi), a differentiation marker expressed in enterocytes. For these differentiation markers, the ECBSH group closely resembled the Conv animals (Figure 2b). Taken together, results suggest that BSH activity influences the expression of genes involved in cell differentiation in immature mice.

To further confirm our findings, we performed immunofluorescence staining for the differentiation marker ALPI and the proliferation marker KI67 (Figure 2c and 2d). We observed a significantly higher percentage of ALPI^+^ cells and a significantly lower percentage of KI67 positive cells in the ECBSH group when compared to the EC and the GF group. Again, the results for the ECBSH treated group were in-line with those obtained for the Conv group. These results are consistent with the RT-qPCR data, which showed a decrease in the mRNA expression levels of KI67 while Alpi was upregulated (Figure 2a, 2c and 2d). As a decrease in proliferation is associated with increased apoptosis levels, we carried out a TUNEL assay on COL tissue samples (Figure 2e). The results showed an increase in the percentage of positive cells in the ECBSH and Conv groups compared to the GF and EC group which is in concordance with the results obtained for KI67 (Figure 2d).

### An unconjugated bile acid mixture represses expression of genes involved in proliferation and stem cell maintenance in colonic organoids

**Figure 3.**
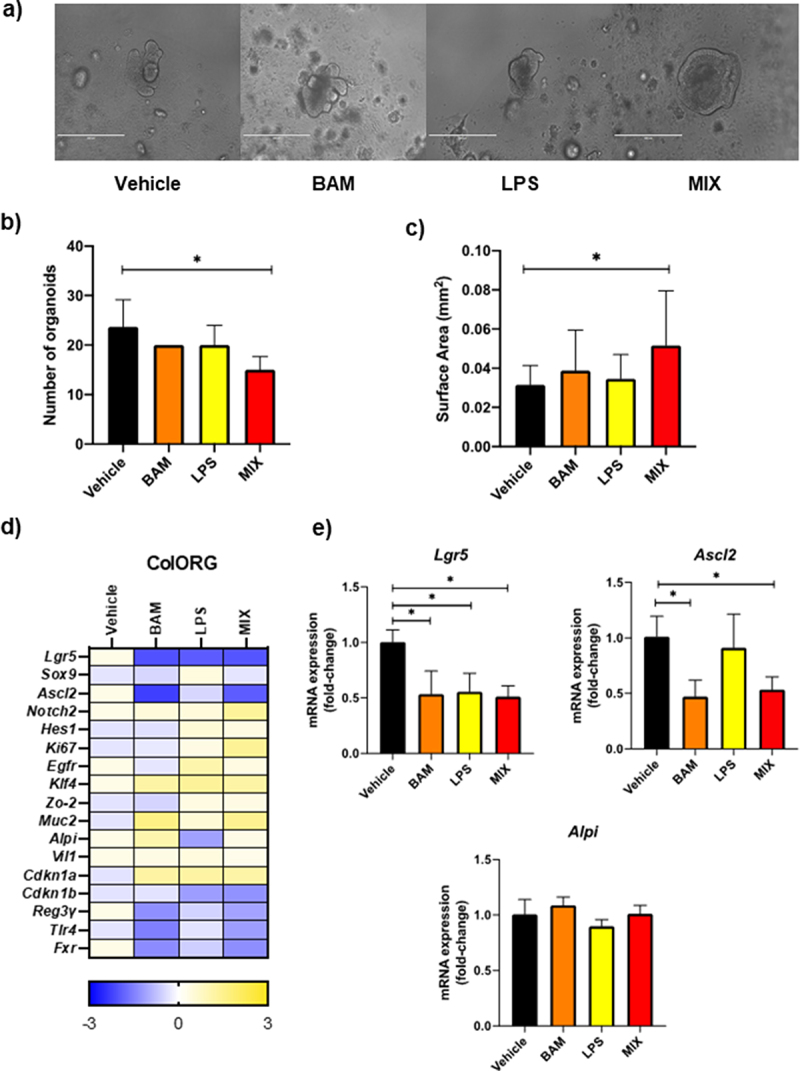
Bile acid signatures influence differentiation of colon organoids. a) Representative pictures of ColORG after 7 days of treatment (bar 400 μm); b) Differences in the number of ColORG after 7 days of treatment; c) Surface area of organoids expressed in mm. All values are represented as mean ± standard deviation. d) ColORG gene expression profile in response to the treatments by qRT-PCR. Represented as the fold-change between the treatment and the vehicle; e) Significant changes in stemness maintenance and differentiation genes by RT-qPCR. All values are represented as the fold change of relative mRNA expression between the treatments and the vehicle. Statistical analyses were conducted using one-way ANOVA and Tukey’s multiple comparison test. All values are represented as mean ± standard deviation. * p < .05.

After identifying the effect of BSH activity in regulating genes involved in differentiation in vivo, we used a three-dimensional *in vitro* model of colon organoids (ColORG) to evaluate whether a mixture of BA could influence the fate of colonic stem cells. ColORG were grown from crypts isolated from female C57BL/6 mice just after weaning and were treated for 7 days with a mixture of unconjugated BA containing cholic acid, ursodeoxycholic acid and chenodeoxycholic acid (BAM) (reflecting the BA produced *in* *vivo* by ECBSH). As *E. coli* (EC) alone demonstrated the ability to promote some effects in GF mice (e.g. on colon length and ALPI expression), albeit to much lower levels than ECBSH, we rationalized that LPS may provide an added signaling function and therefore we included two groups of organoids treated with either LPS or a combination of LPS and BAM (MIX). Neither of the culture conditions affected overall cell viability or proliferation as determined by MTT assay, Annexin V/PI analysis and ClickIT assay by flow cytometry (Supplementary material 7). However, incubation with MIX induced cellular changes to organoids, reducing the total number of ColORG (Figure 3b) and increasing overall ColORG size relative to the vehicle group (Figure 3c).

We analyzed expression of selected genes in treated organoids (Figures 3d and 3e). Results from RT-qPCR analysis showed that, although no changes were observed in *Alpi*, there was a significant reduction in expression of the leucin-rich repeat-containing G-protein couple receptor (*Lgr5*) with all the treatments. Lgr5 is a well-defined marker of self-renewing intestinal stem cells (stemness), which potentiates the canonical Wnt/β-catenin signaling pathway. Interestingly, this repression in *Lgr5* expression levels was accompanied by a reduction in mRNA levels of *Ascl2* only on the BAM and the MIX group, which agrees with the observed results *in vivo* that BSH activity has an impact on intestinal differentiation most likely by influencing such processes. Therefore, in a highly reductionist (organoid) model system (which lacks immune and other mediators) a mixture of BA was able to reduce the expression of key markers of stemness (*Lgr5* and *Ascl2*), albeit that phenotypic changes were not evident unless LPS was also present (MIX group). Further work is ongoing to determine the influences of different BA combinations on these processes. We predict that further inputs, including immune mediators, which are lacking in organoid culture, may be necessary to further influence differentiation markers (including Alpi) that were impacted by BSH *in vivo*.

## Discussion

In the current work, we determined that BSH, a bacterial enzyme widely distributed among the first colonizers of the gut is capable of directing local gene expression in the infant gut and influencing the development and maturation of the GIT. The mechanisms invoke regulation of colonocyte gene expression by altered BA signatures (notably regulation of *Alpi, Ascl2* and *Hes1*), and some of the findings could be recapitulated *in vitro* using a primary ColORG system where bile acids reduced markers of stemness and influenced organoid size.

The seeding and stable colonization of the gut microbiota in early life plays a critical role in the development and maturation of the GIT.^19^ Indeed, disruption of microbiota composition in the infant is associated with long-term systems-wide health effects such as asthma or GIT disorders.^20,21^ Relatively few studies address microbial metabolites in the context of gut development *per se* and more work is necessary to establish how microbe-derived metabolites provide the functional signals that influence host physiological and immune processes in early life.^22,23^

It is well established that the gut of GF mice is poorly developed.^24–26^ Herein we demonstrated that delivery of heterologously expressed BSH activity *in situ* in the gut of infant GF mice can influence physiological and molecular parameters associated with gut development. Analysis of fecal BA profiles in monocolonized gnotobiotic animals confirmed active BSH activity through the production of unconjugated BA in our system.^9^ *In situ* BSH activity significantly reduced COL length and influenced local gene expression patterns resulting in features more closely resembling the Conv group.

To obtain an overview of how BSH activity promotes GIT maturation, we evaluated a panel of genes involved in Wnt and Notch pathways, the main pathways involved in intestinal stem cell maintenance and epithelial differentiation.^27^ *In situ* BSH activity significantly modified the expression of genes involved in Wnt and Notch pathways in a similar manner to expression patterns in Conv animals. We demonstrated that BSH activity significantly represses the expression of the locus encoding the transcription factor *Ascl2* in the COL. ASCL2 is a master regulator of stem cell identity in the GIT.^28^ This was further supported by a reduction in *Aslc2* in a 3D ColORG model exposed to a mixture of unconjugated BAs (BAM) or a combination of LPS and BAM. *Ascl2* is positively regulated by the Wnt pathway, and its expression is restricted to Lgr5^+^ stem cells in the base of the crypt.^29^ When Ascl2 is downregulated, proliferation of stem cells in the base of the crypt is repressed,^29^ which is in line with our findings on *Ascl2* and KI67 expression in the ECBSH-group and on *Lrg5* expression in 3D ColORG exposed to BAM or LPS and BAM.

We also observed a repression of the gene encoding HES1, a basic helix-loop-helix transcription factor, which is a well-established Notch effector, the expression of which is regulated by the canonical Wnt pathway.^30^ Blockade of *Hes1* leads to differentiation of intestinal cells toward an enteroendocrine phenotype and reduces proliferation in crypts.^31^ This reduction in proliferation was again in agreement with the reduction in KI67 by BSH activity which was similar to that observed in the Conv group. A recent study demonstrates that the gut epithelium of neonatal Conv mice exhibits a peak expression of KI67 at week 1 post-partum with a significant reduction at week three.^32^ We show that introduction of BSH activity to the gut of GF mice regulates this reduction in proliferation to levels exhibited in normal (Conv) animals.

Along with the modulation of genes involved in the Wnt and Notch signaling pathways, we observed an increase in the intestinal alkaline phosphatase (*Alpi*) differentiation marker. ALPI is a brush-border enzyme produced following differentiation by enterocytes,^33^ which modulates lipid absorption, detoxification of LPS and other bacterial products, and promotes intestinal homeostasis.^34,35^ Moreover, the repression of *Alpi* has been linked with intestinal inflammatory diseases, microbial dysbiosis, increased permeability and bacterial translocation in newborns.^36^ Our data showed a significant increase in the expression of *Alpi* by BSH activity, which was confirmed by immunofluorescence imaging. Other studies have indicated that an increase in Alpi is associated with improved barrier function.^37^

Whilst our understanding of microbiota composition during early life has increased recently, to date relatively few studies have focused upon how specific microbial-derived metabolites or bacterial activities affect infant development.^19,38,39^ Interestingly a recent analysis of bile acid profiles in human infants demonstrated that bacterial bile acid deconjugation is prevalent during the first two years of life during which secondary bile acid levels are extremely low, suggesting a predominance of microbial BSH activity in early life and a relative lack of microbial conversion to secondary bile acids.^14^ The early appearance of potentially BSH positive bacterial strains derived from the maternal gut microbiota or *Bifidobacterium* species from maternal breast milk in the infant gut^13,14^ and our evidence for the presence of *bsh* genes in the early infant microbiota suggests that this metabolic activity may occur as early as one day postpartum, a period in which the infant gut barrier is underdeveloped.^40^ In contrast the appearance of secondary bile acids appears to correlate with later stages of infant microbiota development when the gut mucosa has already matured.^14^

Here we used a novel approach to evaluate the impact of this single bacterial activity, BSH, in the development and maturation of the GIT in an *in vivo* model. BSH activity promoted a normalization of the gastrointestinal transcriptional landscape toward that of age-matched Conv animals and regulated expression of markers of proliferation (*Ascl1* and *Hes1*) and barrier function (ALPI) (summarized in Supplementary material 8). The work suggests that BA signatures are an important microbiota-derived factor, along with SCFA,^16,17^ in mediating down-regulation of stem cell proliferation and further show that such BA may promote gut development. Importantly these data also suggest an evolutionary paradigm whereby microbial BSH activity influences bacterial niche colonization^7^ and in-turn benefits host gastrointestinal maturation and barrier function.

## Methods

### *In vivo* experimental design and inoculations

Thirty female C57BL/6 mice were maintained in the germ-free animal unit in University College Cork. We appreciate that female GF mice differ from male GF mice in terms of BA physiology (Selwyn paper). Two days after weaning, monocolonization experiments were initiated by oral dosing at a concentration of 10^9^ CFU/50 µL/mice at day 0 and day 3. Inocula were prepared from overnight cultures of *E. coli* MG1655 EC and ECBSH, centrifuged at 4,600 g at 4°C for 15 min, pellets washed in sterile PBS three times and resuspended in sterile PBS to a final concentration of 10^9^ CFU/50 µL. Fecal samples were collected daily to monitor the colonization process by plating on LB agar plates. GF mice feces was consistently sterile as measured by plating. Plasmid retention values were checked after 6 days post-colonization by plating on ampicillin-supplemented LB agar plates. Mice were euthanized and blood was collected in 5 mL tubes. The small intestine (SI) and the colon (COL) lengths were measured before splitting the tissue into collection tubes.

### RNA extraction and cDNA synthesis from tissue samples

Tissue samples from COL, SI and liver (LIV) were collected in 1.5 mL nuclease free tubes containing 1 mL of RNAlater (Sigma) and stored at −80°C until use. mRNA was extracted with the RNA Isolate II mini kit from Bioline following the manufacturer´s recommendations. RNA quality was evaluated using the Nanodrop (Thermo Scientific). Only samples with 260/230 and 260/280 ratios between 1.8 and 2.2 were used for gene expression analyses. cDNA was synthesized using the cDNA kit from NZytech (Portugal) following the manufacturer´s instructions at a concentration of 1000 ng. One negative control for RNA and other for RT were included for every group and tissue.

### Histology

COL and SI samples were collected for histology and immunofluorescence analysis. For histological analysis of crypts samples were processed in Methacarn solution (60% MeOH: 30% Chloroform: 10% acetic acid)^41^ and samples for immunohistochemistry were stored in 10% formalin (details Supplementary material 5).

### Immunofluorescence analyses

COL paraffin sections were cut using a rotary microtome Leica RM2135 and mounted on microscope slide. After overnight incubation at 37°C, sections were deparaffinised with xylene and a series of ethanol solutions to 100% ethanol followed by distilled water bath to rinse slides. An antigen retrieval was performed using 10 mM sodium citrate Buffer (pH 6.0) at 95°C for 30 min. Slides were cooled at room temperature for 20 minutes and washed in PBS. Slides were fixed in 4% PFA for 20 minutes at 4°C, incubated for 1 h at RT in blocking buffer (10% serum in PBS-Tween) and incubated with the primary antibody (diluted in 1% Bovine serum albumin and PBS-Tween) over night at 4°C: KI67 or Alkaline Phosphatase (ALPI) (Rabbit anti-mouse KI67, ab15580, abcam, 1:200/ Rabbit anti-mouse ALPI, PA575668, invitrogen,1:200). The slides were washed in PBS three times for 5 mins and were then incubated with the secondary antibody (goat anti rabbit IgG Alexa Fluor488, 2 mg/ml, Invitrogen, 1:1000, diluted in 1% Bovine serum albumin and PBS-Tween) for 1 h at RT in the dark. After washings, slides were stained with DAPI and cover slipped using mounting media. Sections were observed with a confocal laser microscope (Nikon D-Eclipse) using x100 magnification and pictures were taken. Pictures were analyzed using NIS-Elements BR 3.0 software.

### Determination of apoptosis by TUNEL assay

Apoptosis was detected in COL samples using a kit from Abcam (Cat # ab206386; Cambridge, United Kingdom) according to the manufacturer’s instructions. Sections were observed with a bright field microscope confocal laser microscope (Olympus BX43) using x40 magnification and pictures were taken. Pictures were analyzed using NIS-Elements BR 3.0 software.

### Determination of bile acid and short-chain fatty acid profiles

Bile acid moieties and short-chain fatty acid profiles were determined as outlined in Supplementary material 3.

### Crypt isolation, organoid culture and treatments

Colon crypt isolation was performed using colon tissues isolated from four 3-week old female C57BL/6 mice as described by Sato et al.,^42^ with some modifications (see Supplementary material 7).

### RNA isolation and RT-qPCR

For organoids, BME was disrupted with 5 mM EDTA in PBS, collected in 1.5 mL tubes and placed in ice for 1 h. and centrifuged at 200 x g for 5 min at 4°C. Organoids were then washed three times with cold DPBS and then resuspended in lysis buffer. mRNA extraction was done using the RNeasy micro kit from Qiagen following manufacturer´s instructions. For tissues, RNA isolation was carried out as described previously.^41^ Only samples with 260/230 and 260/280 ratios between 1.8 and 2.2 were used for gene expression analyses. cDNA was synthesized using the cDNA kit from NZytech (Portugal) following the manufacturer´s instructions at a concentration of 100 ng. One negative control for RNA and other for RT were included for each treatment. Transcriptomic analysis was done by RT-qPCR using LightCycler® 480 Probes Master (Roche) with the Universal Probe Library from Roche. Primers are outlined in Supplementary material 6, *ACTB* was used as a housekeeping gene control. The amplification setup used was 45 runs in 384-well plates with the MonoColor hydrolysis probe detection format.

### Statistical analysis

All data are presented as mean values ± SD Statistical analyses were performed with GraphPad Prism software version 8.4.3 (GraphPad Software, La Jolla, CA). Normal distribution of the variables was assessed by the Shapiro-Wilk test. Data were analyzed using the one-way analysis of variance with Tukey’s test for multiple comparisons in those variables following normal distribution and the Kruskal-Wallis one-way analysis of variance on ranks followed by Dunn’s post hoc analysis for the intergroup differences. A *p* value <.05 was considered significant.

## Supplementary Material

Supplemental Material

## Data Availability

All data are available on Zenodo at the following link https://doi.org/10.5281/zenodo.6610545.

## References

[1] Fanaro S, Chierici R, Guerrini P, Vigi V. Intestinal microflora in early infancy: composition and development. Acta Paediatr Suppl. 2003;91(441):48–12. doi:10.1111/j.1651-2227.2003.tb00646.x. 14599042

[2] Fulde M, Hornef MW. Maturation of the enteric mucosal innate immune system during the postnatal period. Immunol Rev. 2014;260(1):21–34. doi:10.1111/imr.12190. 24942679

[3] Gasaly N, de Vos P, Hermoso MA. Impact of bacterial metabolites on gut barrier function and host immunity: a focus on bacterial metabolism and its relevance for intestinal inflammation. Front Immunol. 2021;12:658354. doi:10.3389/fimmu.2021.658354. 34122415 PMC8187770

[4] Sun R, Xu C, Feng B, Gao X, Liu Z. Critical roles of bile acids in regulating intestinal mucosal immune responses. Therap Adv Gastroenterol. 2021;14:17562848211018098. doi:10.1177/17562848211018098. PMC8165529 34104213

[5] Long SL, Gahan CGM, Joyce SA. Interactions between gut bacteria and bile in health and disease. Mol Aspects Med. 2017;56:54–65. doi:10.1016/j.mam.2017.06.002. 28602676

[6] Begley M, Gahan CG, Hill C. The interaction between bacteria and bile. FEMS Microbiol Rev. 2005;29(4):625–651. doi:10.1016/j.femsre.2004.09.003. 16102595

[7] Jones BV, Begley M, Hill C, Gahan CG, Marchesi JR. Functional and comparative metagenomic analysis of bile salt hydrolase activity in the human gut microbiome. Proc Natl Acad Sci U S A. 2008;105(36):13580–13585. doi:10.1073/pnas.0804437105. 18757757 PMC2533232

[8] Seedorf H, Griffin NW, Ridaura VK, Reyes A, Cheng J, Rey FE, Smith M, Simon G, Scheffrahn R, Woebken D, et al. Bacteria from diverse habitats colonize and compete in the mouse gut. Cell. 2014;159(2):253–266. doi:10.1016/j.cell.2014.09.008. 25284151 PMC4194163

[9] Joyce SA, MacSharry J, Casey PG, Kinsella M, Murphy EF, Shanahan F, Hill C, Gahan CGM. Regulation of host weight gain and lipid metabolism by bacterial bile acid modification in the gut. Proc Natl Acad Sci U S A. 2014;111(20):7421–7426. doi:10.1073/pnas.1323599111. 24799697 PMC4034235

[10] Inagaki T, Moschetta A, Lee YK, Peng L, Zhao G, Downes M, Yu RT, Shelton JM, Richardson JA, Repa JJ, et al. Regulation of antibacterial defense in the small intestine by the nuclear bile acid receptor. Proc Natl Acad Sci U S A. 2006;103(10):3920–3925. doi:10.1073/pnas.0509592103. 16473946 PMC1450165

[11] Sayin SI, Wahlstrom A, Felin J, Jantti S, Marschall HU, Bamberg K, Angelin B, Hyötyläinen T, Orešič M, Bäckhed F, et al. Gut microbiota regulates bile acid metabolism by reducing the levels of tauro-beta-muricholic acid, a naturally occurring FXR antagonist. Cell Metab. 2013;17(2):225–235. doi:10.1016/j.cmet.2013.01.003. 23395169

[12] van Best N, Rolle-Kampczyk U, Schaap F G, Basic M, Olde Damink S W, Bleich A, Savelkoul P H, von Bergen M, Penders J, Hornef M W, et al. Bile acids drive the newborn's gut microbiota maturation. Nat Commun. 2020;11(1):3692. doi: 10.1038/s41467-020-17183-8. 32703946 PMC7378201

[13] Ferretti P, Pasolli E, Tett A, Asnicar F, Gorfer V, Fedi S, Armanini F, Truong DT, Manara S, Zolfo M, et al. Mother-to-infant microbial transmission from different body sites shapes the developing infant gut microbiome. Cell Host Microbe. 2018;24(1):133–45 e5. doi:10.1016/j.chom.2018.06.005. 30001516 PMC6716579

[14] Tanaka M, Sanefuji M, Morokuma S, Yoden M, Momoda R, Sonomoto K, Ogawa M, Kato K, Nakayama J. The association between gut microbiota development and maturation of intestinal bile acid metabolism in the first 3 y of healthy Japanese infants. Gut Microbes. 2020;11(2):205–216. doi:10.1080/19490976.2019.1650997. 31550982 PMC7053967

[15] Selwyn F Pavithra, Csanaky I L, Zhang Y, Klaassen C D. Importance of Large Intestine in Regulating Bile Acids and Glucagon-Like Peptide-1 in Germ-Free Mice. Drug Metab Dispos. 2015;43(10):1544–56. doi: 10.1124/dmd.115.065276. 26199423 PMC4576674

[16] Kaiko GE, Ryu SH, Koues OI, Collins PL, Solnica-Krezel L, Pearce EJ, Pearce EL, Oltz EM, Stappenbeck TS. The colonic crypt protects stem cells from microbiota-derived metabolites. Cell. 2016;165(7):1708–1720. doi:10.1016/j.cell.2016.05.018. 27264604 PMC5026192

[17] Pearce SC, Weber GJ, van Sambeek DM, Soares JW, Racicot K, Breault DT, van Sambeek DM. Intestinal enteroids recapitulate the effects of short-chain fatty acids on the intestinal epithelium. PLoS One. 2020;15(4):e0230231. doi:10.1371/journal.pone.0230231. 32240190 PMC7117711

[18] Foley SE, Tuohy C, Dunford M, Grey MJ, De Luca H, Cawley C, Szabady RL, Maldonado-Contreras A, Houghton JM, Ward DV, et al. Gut microbiota regulation of P-glycoprotein in the intestinal epithelium in maintenance of homeostasis. Microbiome. 2021;9(1):183. doi:10.1186/s40168-021-01137-3. 34493329 PMC8425172

[19] Hill DR, Huang S, Nagy MS, Yadagiri VK, Fields C, Mukherjee D, Bons B, Dedhia PH, Chin AM, Tsai Y-H, et al. Bacterial colonization stimulates a complex physiological response in the immature human intestinal epithelium. Elife. 2017;6. doi:10.7554/eLife.29132. PMC5711377 29110754

[20] Cereta AD, Oliveira VR, Costa IP, Guimaraes LL, Afonso JPR, Fonseca AL, Sousa ARTD, Silva GAM, Mello DACPG, Oliveira LVFD, et al. Early Life Microbial Exposure and Immunity Training Effects on Asthma Development and Progression. Front Med. 2021;8:662262. doi:10.3389/fmed.2021.662262. PMC8241902 34222279

[21] Round JL, Mazmanian SK. The gut microbiota shapes intestinal immune responses during health and disease. Nat Rev Immunol. 2009;9(5):313–323. doi:10.1038/nri2515. 19343057 PMC4095778

[22] Milani C, Duranti S, Bottacini F, Casey E, Turroni F, Mahony J, Belzer C, Delgado Palacio S, Arboleya Montes S, Mancabelli L, Lugli GA. The first microbial colonizers of the human gut: composition, activities, and health implications of the infant gut microbiota. Microbiol Mol Biol Rev. 2017;81:e00036–17. 10.1128/MMBR.00036-17 PMC5706746 29118049

[23] Sittipo P, Shim JW, Lee YK. Microbial Metabolites determine host health and the status of some diseases. Int J Mol Sci. 2019;20:5296. 31653062 10.3390/ijms20215296 PMC6862038

[24] Al-Asmakh M, Zadjali F. Use of germ-free animal models in microbiota-related research. J Microbiol Biotechnol. 2015;25(10):1583–1588. doi:10.4014/jmb.1501.01039. 26032361

[25] Sommer F, Backhed F. The gut microbiota–masters of host development and physiology. Nat Rev Microbiol. 2013;11(4):227–238. doi:10.1038/nrmicro2974. 23435359

[26] Slezak K, Krupova Z, Rabot S, Loh G, Levenez F, Descamps A, Lepage P, Doré J, Bellier S, Blaut M, et al. Association of germ-free mice with a simplified human intestinal microbiota results in a shortened intestine. Gut Microbes. 2014;5(2):176–182. doi:10.4161/gmic.28203. 24637599 PMC4063842

[27] Gracz AD, Magness ST. Defining hierarchies of stemness in the intestine: evidence from biomarkers and regulatory pathways. Am J Physiol Gastrointest Liver Physiol. 2014;307(3):G260–73. doi:10.1152/ajpgi.00066.2014. 24924746 PMC4121637

[28] Schuijers J, Junker JP, Mokry M, Hatzis P, Koo BK, Sasselli V, Van der flier L, Cuppen E, Van oudenaarden A, Clevers H, et al. Ascl2 acts as an R-spondin/Wnt-responsive switch to control stemness in intestinal crypts. Cell Stem Cell. 2015;16(2):158–170. doi:10.1016/j.stem.2014.12.006. 25620640

[29] van der Flier LG, van Gijn ME, Hatzis P, Kujala P, Haegebarth A, Stange DE, Begthel H, van den Born M, Guryev V, Oving I, et al. Transcription factor achaete scute-like 2 controls intestinal stem cell fate. Cell. 2009;136(5):903–912. doi:10.1016/j.cell.2009.01.031. 19269367

[30] Goto N, Ueo T, Fukuda A, Kawada K, Sakai Y, Miyoshi H, Taketo MM, Chiba T, Seno H. Distinct roles of HES1 in normal stem cells and tumor stem-like cells of the intestine. Cancer Res. 2017;77(13):3442–3454. doi:10.1158/0008-5472.CAN-16-3192. 28536281

[31] Ueo T, Imayoshi I, Kobayashi T, Ohtsuka T, Seno H, Nakase H, Chiba T, Kageyama R. The role of Hes genes in intestinal development, homeostasis and tumor formation. Development. 2012;139(6):1071–1082. doi:10.1242/dev.069070. 22318232

[32] Stanford AH, Gong H, Noonan M, Lewis AN, Gong Q, Lanik WE, Hsieh JJ, Lueschow SR, Frey MR, Good M, et al. A direct comparison of mouse and human intestinal development using epithelial gene expression patterns. Pediatr Res. 2020;88(1):66–76. doi:10.1038/s41390-019-0472-y. 31242501 PMC6930976

[33] Sussman NL, Eliakim R, Rubin D, Perlmutter DH, DeSchryver-Kecskemeti K, Alpers DH. Intestinal alkaline phosphatase is secreted bidirectionally from villous enterocytes. Am J Physiol. 1989;257(1 Pt 1):G14–23. doi:10.1152/ajpgi.1989.257.1.G14. 2546440

[34] Singh SB, Carroll-Portillo A, Coffman C, Ritz NL, Lin HC. Intestinal alkaline phosphatase exerts anti-inflammatory effects against lipopolysaccharide by inducing autophagy. Sci Rep. 2020;10(1):3107. doi:10.1038/s41598-020-59474-6. 32080230 PMC7033233

[35] Lalles JP. Intestinal alkaline phosphatase: novel functions and protective effects. Nutr Rev. 2014;72(2):82–94. doi:10.1111/nure.12082. 24506153

[36] Fawley J, Koehler S, Cabrera S, Lam V, Fredrich K, Hessner M, Salzman N, Gourlay D. Intestinal alkaline phosphatase deficiency leads to dysbiosis and bacterial translocation in the newborn intestine. J Surg Res. 2017;218:35–42. doi:10.1016/j.jss.2017.03.049. 28985873

[37] Liu W, Hu D, Huo H, Zhang W, Adiliaghdam F, Morrison S, Ramirez JM, Gul SS, Hamarneh SR, Hodin RA, et al. Intestinal alkaline phosphatase regulates tight junction protein levels. J Am Coll Surg. 2016;222(6):1009–1017. doi:10.1016/j.jamcollsurg.2015.12.006. 27106638 PMC5684582

[38] Dougherty MW, Kudin O, Muhlbauer M, Neu J, Gharaibeh RZ, Jobin C. Gut microbiota maturation during early human life induces enterocyte proliferation via microbial metabolites. BMC Microbiol. 2020;20(1):205. doi:10.1186/s12866-020-01892-7. 32652929 PMC7353703

[39] Gomez de Aguero M, Ganal-Vonarburg SC, Fuhrer T, Rupp S, Uchimura Y, Li H, Steinert A, Heikenwalder M, Hapfelmeier S, Sauer U, et al. The maternal microbiota drives early postnatal innate immune development. Science. 2016;351(6279):1296–1302. doi:10.1126/science.aad2571. 26989247

[40] Halpern MD, Denning PW. The role of intestinal epithelial barrier function in the development of NEC. Tissue Barriers. 2015;3(1–2):e1000707. doi:10.1080/21688370.2014.1000707. 25927016 PMC4389790

[41] Las Heras V, Clooney AG, Ryan FJ, Cabrera-Rubio R, Casey PG, Hueston CM, Pinheiro J, Rudkin JK, Melgar S, Cotter PD, et al. Short-term consumption of a high-fat diet increases host susceptibility to Listeria monocytogenes infection. Microbiome. 2019;7(1):7. doi:10.1186/s40168-019-0621-x. 30658700 PMC6339339

[42] Sato T, Stange DE, Ferrante M, Vries RG, Van Es JH, Van den Brink S, van Houdt WJ, Pronk A, van Gorp J, Siersema PD, et al. Long-term expansion of epithelial organoids from human colon, adenoma, adenocarcinoma, and Barrett’s epithelium. Gastroenterology. 2011;141(5):1762–1772. doi:10.1053/j.gastro.2011.07.050. 21889923

