# Supplementary material for: Microbial bile salt hydrolase activity influences gene expression profiles and gastrointestinal maturation in infant mice
Source: Gut Microbes. 2022 Nov 24;14(1):2149023. doi: 10.1080/19490976.2022.2149023 (PMC9704388; doi:10.1080/19490976.2022.2149023)
Supplement: Supplemental Material [file KGMI_A_2149023_SM9587.zip › Núñez-Sánchez Supp Material 1 (1).pdf]

Supplementary material 1 –
in silico analysis of BSH in human datasets

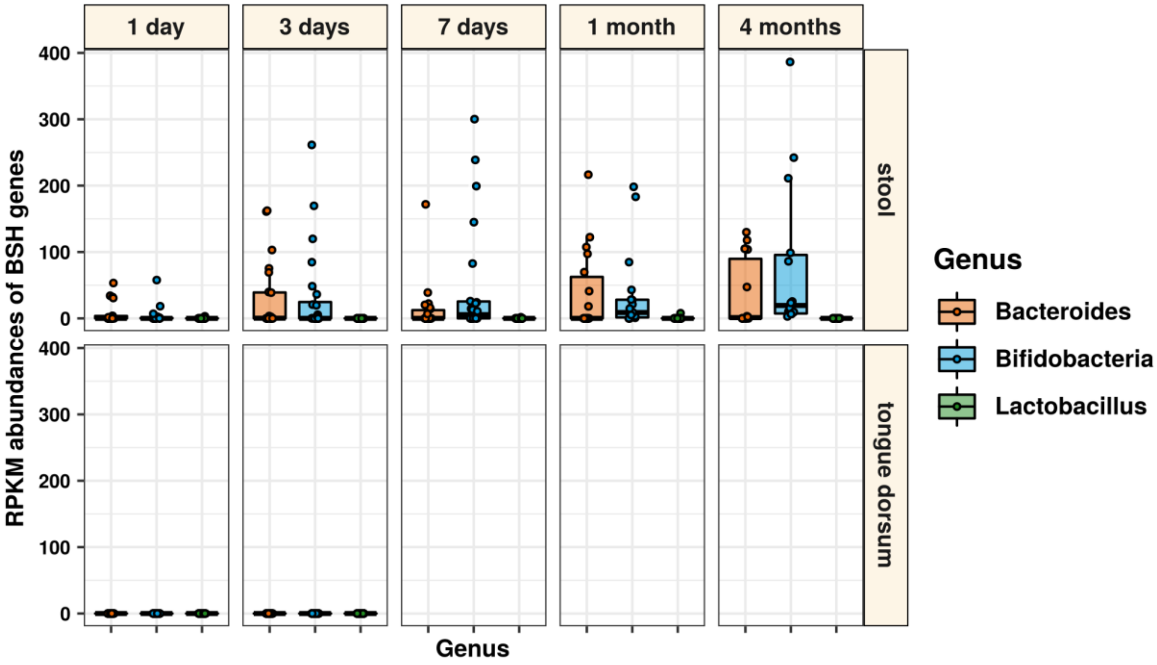

Quantification of bile salt hydrolases (BSH) in an infant metagenomic cohort

ShortBRED (v0.9.4) was used to quantify composition and abundance of BSH genes (*Kaminski et al*., 2015). We mainly focused on a total of 31 BSH protein sequences that belong to different species from *Lactobacillus* (n=11), *Bacteroides* (n=10), and *Bifidobacteria* (n=10) genus (Table S1). ShortBRED specific unique marker sequences were generated from selected BSH protein sequences with following parameter (--clustid 0.85 --ref Uniref90.fasta) and subsequently used to quantify BSH genes in metagenomic samples using default parameters. We specifically focused on an infant metagenomic cohort from *Ferretti et al*. (2018) for which multiple infant samples (stool and tongue dorsum) were collected in the first four months of life. Briefly, raw fastq files were downloaded using the fastq-dump utility script in SRA Toolkit. Quality filtering and host decontamination of raw sequencing data was carried out using Kneaddata tool with default parameters. We processed 136 valid SRA runs (from Infants) associated with the NCBI Bio project PRJNA352475 that consisted of an average of 20.2 million quality filtered reads. Individual marker level abundance in reads per kilobase per million reads (RPKM) was further grouped by its genus level assignment.

References

Ferretti P, Pasolli E, Tett A, Asnicar F, Gorfer V, Fedi S, et al. Mother-to-Infant Microbial Transmission from Different Body Sites Shapes the Developing Infant Gut Microbiome. Cell Host Microbe 2018; 24:133-45 e5.

Kaminski J, Gibson MK, Franzosa EA, Segata N, Dantas G, Huttenhower C: High-Specificity Targeted Functional Profiling in Microbial Communities with ShortBRED. PLoS Comput Biol 2015, 11:e1004557.

Supplementary Material 1 cont…

Supplementary Table S1. Metagenomic query sequences used in previous figure.

NCBI accessions	Genus	Species/ Strains
WP_003546965.1	Lactobacillus	Lactobacillus acidophilus NCFM
WP_010690294.1	Lactobacillus	Lactobacillus animalis
AEB72500.1	Lactobacillus	Lactobacillus buchneri NRRL B-30929
AEZ06356.1	Lactobacillus	Lactobacillus fermentum NCDO 394
ABJ59469.1	Lactobacillus	Lactobacillus gasseri ATC 33323
AAS09178.1	Lactobacillus	Lactobacillus johnsonii NCC533
ADN97280.1	Lactobacillus	Lactobacillus plantarum ST-III
EDX41535.1	Lactobacillus	Lactobacillus reuteri 100-23
ACL98204.1	Lactobacillus	Lactobacillus salivarius JCM1046
WP_056974571.1	Lactobacillus	Lactobacillus vaginalis
CCC80500.1	Lactobacillus	Lactobacillus plantarum WCFS1
WP_195394019.1	Bifidobacteria	Bifidobacteria adolescentis
WP_045919813.1	Bifidobacteria	Bifidobacteria angulatum
WP_130079430.1	Bifidobacteria	Bifidobacteria animalis
WP_053824989.1	Bifidobacteria	Bifidobacteria bifidum
AUD87005.1	Bifidobacteria	Bifidobacteria breve
WP_039198334.1	Bifidobacteria	Bifidobacteria catenulatum
WP_006294150.1	Bifidobacteria	Bifidobacteria gallicum
WP_032743741.1	Bifidobacteria	Bifidobacteria longum
WP_209107517.1	Bifidobacteria	Bifidobacteria pseudocatenulatum
WP_026646013.1	Bifidobacteria	Bifidobacteria ruminatium
EDU99086.1	Bacteroides	Bacteroides coprocola DSM 17136
EEO44970.1	Bacteroides	Bacteroides dorei 5_1_36/D4
EEC53612.1	Bacteroides	Bacteroides eggerthii DSM 20697
BAD50536.1	Bacteroides	Bacteroides fragilis YCH46
EDV03500.1	Bacteroides	Bacteroides intestinalis DSM 17393
EDO11390.1	Bacteroides	Bacteroides ovatus ATCC8483
AAO77193.1	Bacteroides	Bacteroides thetaoitaomicron VPI_5482
EDO55730.1	Bacteroides	Bacteroides uniformis ATCC 8492
ABR41596.1	Bacteroides	Bacteroides vulgatus ATCC 8482
CDM05097.1	Bacteroides	Bacteroides xylamisolvens SD CC 1b
