# Supplementary material for: Microbial bile salt hydrolase activity influences gene expression profiles and gastrointestinal maturation in infant mice
Source: Gut Microbes. 2022 Nov 24;14(1):2149023. doi: 10.1080/19490976.2022.2149023 (PMC9704388; doi:10.1080/19490976.2022.2149023)
Supplement: Supplemental Material [file KGMI_A_2149023_SM9587.zip › Núñez-Sánchez Supp Material 2.pdf]

Supplementary material 2
Construction of *E. coli* BSH

a)

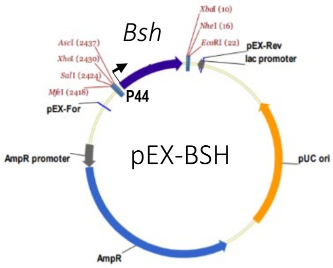

b)

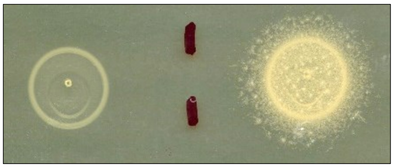

E. coli *E. coli* BSH

E. coli BSH Strain construction a) To construct an *Escherichia coli* strain expressing bile salt hydrolase (BSH) using a multi-copy plasmid and strong promoter, the sequence corresponding to the *L. salivarius* JCM1046 BSH1 (accession number FJ591081.1) was synthesised downstream of the P44 promoter into the standard cloning vector pEX-A128 (Eurofins Genomics) to generate pEX-BSH. The plasmid was then purified and individually transformed into the commensal *E. coli* MG1655 (courtesy of the Yale Culture Collection). b) 5µl of overnight cultures were plated onto LB agar plates containing 5mM TDCA and incubated for 48 hours at 37° C.
