# Supplementary material for: Microbial bile salt hydrolase activity influences gene expression profiles and gastrointestinal maturation in infant mice
Source: Gut Microbes. 2022 Nov 24;14(1):2149023. doi: 10.1080/19490976.2022.2149023 (PMC9704388; doi:10.1080/19490976.2022.2149023)
Supplement: Supplemental Material [file KGMI_A_2149023_SM9587.zip › Núñez-Sánchez Supp Material 3.pdf]

Supplementary material 3a –
Bile acid analysis in mouse faeces

Supplementary Table S2. Bile acids quantified in faecal samples:

Bile Acids	GF	EC	ECBSH	Conv	P value
Total BA	2343.47 ± 1186.83<br>(787.80 – 3491.37)	4174.40 ± 1494.83<br>(1811.51 – 5572.16)	3402.72 ± 2930.61<br>(651.71 – 7029.69)	4387.48 ± 2725.18<br>(709.87 – 7036.98)	NS
Unconjugated BA	24.91 ± 6.44 ^{a}<br>(14.96 – 29.73)	33.27 ± 7.13 ^{a}<br>(25.23 – 43.44)	363.34 ± 232.81 ^{a}<br>(119.43 – 601.38)	4266.61 ± 2705.08 ^{b}<br>(692.06 – 6944.07)	< 0.001
Conjugated BA	2318.57 ± 1188.41^{a,b}<br>(759.44 – 3469.54)	4141.13 ± 1489.97^{a}<br>(1786.28 – 5528.72)	3039.37 ± 2704.53 ^{a,b}<br>(532.28 – 6428.31)	120.87 ± 50.39 ^{b}<br>(80.81 – 193.08)	0.022
Primary BA	2342.96 ± 1186.79<br>(787.37 – 3490.86)	4173.85 ± 1494.77<br>(1811.12 – 5571.47)	3401.87 ± 2930.64<br>(651.33 – 7028.52)	3364.95 ± 2075.66<br>(671.00 – 5467.13)	NS
Unconjugated	24.49 ± 6.35^{a}<br>(14.66 – 29.20)	32.90 ± 7.03^{a}<br>(24.96 – 42.98)	362.56 ± 232.94^{a}<br>(119.07 – 600.21)	3253.75 ± 2059.11 ^{b}<br>(593.57 – 5378.20)	< 0.001
CA	10.99 ± 0.16^{a}<br>(10.81 – 11.13)	13.29 ± 2.26^{a,b}<br>(10.68 – 16.04)	198.96 ± 128.40^{b}<br>(66.39 – 325.5)	169.07 ± 197.71^{a,b}<br>(35.19 – 459.30)	< 0.001
CDCA	0.11 ± 0.02^{a}<br>(0.08 – 0.13)	0.23 ± 0.08^{a,b}<br>(0.18 – 0.36)	6.96 ± 5.23^{b}<br>(1.68 – 12.81)	12.37 ± 14.11^{b}<br>(1.35 – 32.94)	< 0.001
α-MCA	6.02 ± 0.91^{a}<br>(4.73 – 6.74)	5.68 ± 1.21^{a}<br>(4.25 – 6.69)	7.10 ± 1.95^{a}<br>(4.31 – 9.24)	1801.73 ± 1158.03^{b}<br>(445.3 – 3254)	< 0.001
β-MCA	11.36 ± 2.00^{a}<br>(9.43 – 14.30)	13.21 ± 3.75^{a}<br>(9.44 – 19.34)	143.69 ± 96.05^{a,b}<br>(42.01 – 244.2)	1248.94 ± 855.00^{b}<br>(107.6 – 2026)	0.003
UDCA	0.43 ± 0.03^{a}<br>(0.37 – 0.45)	0.49 ± 0.10^{a}<br>(0.37 – 0.62)	5.84 ± 3.91^{a,b}<br>(1.75 – 10.13)	21.64 ± 15.58^{b}<br>(4.15 – 39.34)	0.003
Conjugated	2318.46 ± 1188.36^{a,b}<br>(759.40 – 3469.37)	4140.95 ± 1489.92^{a}<br>(1786.16 – 5528.49)	3039.31 ± 2704.53^{a,b}<br>(532.26 – 6428.31)	111.21 ± 41.00^{b}<br>(77.43 – 169.53)	0.022
TCA	683.97 ± 361.91^{a,b}<br>(222.9 – 1030)	1309.19 ± 514.81^{a}<br>(526.3 – 1732)	925.05 ± 831.43^{a,b}<br>(171.1 – 1952)	40.05 ± 14.37^{b}<br>(28.22 – 58.23)	0.022
TCDCA	30.84 ± 17.74^{a}<br>(10.53 – 53.38)	76.44 ± 10.75^{b}<br>(67.55 – 90.35)	33.11 ± 35.11^{a}<br>(3.74 – 87.29)	4.39 ± 2.54^{a}<br>(2.57 – 8.14)	0.003
TMCA	1568.88 ± 790.41^{a,b}<br>(517.8 – 2339)	2704.88 ± 948.39^{a}<br>(1238 – 3786)	2041.08 ± 1800.48^{a,b}<br>(352.4 – 4289)	63.63 ± 23.62^{b}<br>(44.34 – 98.04)	0.023
TUDCA	33.64 ± 20.36^{a,b}<br>(7.74 – 54.95)	64.14 ± 29.02^{a}<br>(21.08 – 100.4)	38.83 ± 40.38^{a,b}<br>(4.60 – 97.29)	2.17 ± 0.97^{b}<br>(1.28 – 3.37)	0.035
GCA	0.12 ± 0.06<br>(0.06 – 0.20)	0.23 ± 0.09<br>(0.10 – 0.35)	0.19 ± 0.15<br>(0.05 – 0.38)	0.14 ± 0.09<br>(0.06 – 0.28)	NS
GCDCA	ND	0.01 ± 0.00<br>(0.01 – 0.01)	0.01 ± 0.01<br>(0.00 – 0.01)	0.01 ± 0.01<br>(0.00 – 0.02)	NA
GMCA	0.89 ± 0.41<br>(0.29 – 1.37)	1.35 ± 0.43<br>(0.70 – 1.84)	1.02 ± 0.85<br>(0.25 – 2.31)	0.82 ± 0.61<br>(0.08 – 1.44)	NS
GUDCA	0.01 ± 0.01<br>(0.00 – 0.02)	0.01 ± 0.00<br>(0.01 – 0.01)	0.01 ± 0.01<br>(0.00 – 0.03)	0.01 ± 0.01<br>(0.00 – 0.02)	NS
Secondary BA	0.51 ± 0.09^{a}<br>(0.41 – 0.60)	0.55 ± 0.14^{a}<br>(0.39 – 0.71)	0.85 ± 0.62^{a}<br>(0.32 – 1.78)	1350.41 ± 195.09^{b}<br>(1196.55 – 1569.85)	< 0.001
Unconjugated	0.40 ± 0.09^{a}<br>(0.30 – 0.53)	0.37 ± 0.14^{a}<br>(0.21 – 0.56)	0.78 ± 0.64^{a}<br>(0.20 – 1.73)	1338.65 ± 200.65^{b}<br>(1188.81 – 1565.87)	< 0.001
DCA	0.34 ± 0.09^{a}<br>(0.29 – 0.51)	0.35 ± 0.14^{a}<br>(0.19 – 0.54)	0.75 ± 0.63^{a}<br>(0.19 – 1.67)	1243.50 ± 168.12^{b}<br>(1117 – 1434)	< 0.001
LCA	0.02 ± 0.01^{a}<br>(0.01 – 0.02)	0.02 ± 0.00^{a,b}<br>(0.02 – 0.02)	0.03 ± 0.02^{a,b}<br>(0.01 – 0.06)	32.16 ± 5.15^{b}<br>(26.89 – 39.01)	0.013
HDCA	ND	ND	ND	48.08 ± 34.81<br>(8.60 – 92.67)	NA
Conjugated	0.11 ± 0.05^{a}<br>(0.04 – 0.17)	0.18 ± 0.07^{a}<br>(0.12 – 0.27)	0.08 ± 0.05^{a}<br>(0.02 – 0.12)	11.75 ± 10.38^{b}<br>(3.98 – 23.55)	0.003
TDCA	ND	ND	ND	6.79 ± 7.21<br>(2.27 – 17.44)	NA
TLCA	0.11 ± 0.05<br>(0.04 – 0.17)	0.18 ± 0.07<br>(0.12 – 0.27)	0.08 ± 0.05<br>(0.02 – 0.12)	0.44 ± 0.50<br>(0.07 – 1.15)	NS
THDCA	ND	ND	ND	2.24 ± 1.73<br>(0.99 – 4.70)	NA
GDCA	ND	ND	ND	0.16 ± 0.13<br>(0.03 – 0.31)	NA
GLCA	ND	ND	ND	0.02 ± 0.01<br>(0.01 – 0.02)	NA
GHDCA	ND	ND	ND	0.02 ± 0.01<br>(0.01 – 0.03)	NA

Legend on next page

Legend Table S2: Values are expressed as nmol per gram (mean ± SD, and range of quantified metabolites). *P values* were calculated using ANOVA test in those parameters with a normal distribution (Total BA, Unconjugated BA, Conjugated BA, Primary BA, Unconjugated primary BA, Conjugated primary BA, Secondary BA, Unconjugated secondary BA, Conjugated secondary BA, DCA, α-MCA, TCA, TCDCA, TLCA, GCA, TUDCA, TMCA and GMCA), whereas *P* values for parameters without a normal distribution (CA, CDCA, LCA, UDCA, β-MCA, and GUDCA) were calculated using a Kruskal-Wallis test (considering *P* < 0.05 significant), followed by a Tukey or Dunn's post hoc analysis for the intergroup differences test respectively in those parameters with a significant *P value*. Different superscript letters indicate statistically significant difference at *P* < 0.05 within each row between the groups. BA, Bile acids; CA, cholic acid; CDCA, chenodeoxycholic acid; α-MCA, α-muricholic acid; β-MCA, β-muricholic acid; TCA, taurocholic acid; TCDCA, taurochenodeoxycholic acid; TMCA, tauromuricholic acid; GCA, glycocholic acid; GCDCA, glycochenodeoxycholic acid; GMCA, glycomuricholic acid; DCA, deoxycholic acid; LCA, lithocholic acid; UDCA, ursodeoxycholic acid; HDCA, hyodeoxycholic acid; TDCA, taurodeoxycholic acid; TLCA, taurolithocholic acid; TUDCA, tauroursodeoxycholic acid; THDCA, taurohyodeoxycholic acid; GDCA, glycodeodeoxycholic acid; GLCA, glycolithocholic acid; GUDCA, glycoursodeoxycholic acid; GHDCA, glycohyodeoxycholic acid; ND, Non detected (below limit of detection); NA, non-applicable; NS, not significant.

Supplementary material 3a –
Bile acid analysis in mouse faeces

Materials and Methods Bile acid analysis

Samples (10 mg faecal matter) were placed in a 2 mL Eppendorf tube and mixed with 20 µL of internal standard solution (Cholic acid-d4 and Taurocholic Acid-d5), 800 µL of 0.1M NaOH and a steel bead. Samples were homogenized with a bullet blender for 3 minutes at speed 8 and vortexed for 5 minutes and incubated at 60 ºC for 1 hour. Afterwards, a volume of 600 µL of water was added and the samples were centrifuged for 10 minutes at 15000 rpm and 4ºC. Supernatants were loaded to a SPE cartridge (Oasis HLB 30 mg sorbent) previously conditioned with 1 mL of methanol and 1 mL of water. Cartridges were washed with 1 mL of water, 1 mL of hexane and 1 mL of water. Then, cartridges were dried under high vacuum and compounds were eluted with 500 µL of methanol twice. The elutes were evaporated to dryness in a SpeedVac at 45 ºC. and reconstituted with 100 µL of methanol for their analysis by UHPLC-MS/MS.

The quantification of 15 bile acids (see Supplementary Methods) in 80 mouse faecal samples were performed by ultra-high performance liquid chromatography coupled to triple quadrupole mass spectrometry using an UHPLC 1290 Infinity II Series coupled to a QqQ/MS 6490 Series (Agilent Technologies, Sta. Clara, CA, USA).

The chromatographic separation was performed with a gradient elution on a Kinetex EVO C18 (150 x 2.1 mm, 2.6 µm) (Phenomenex, Torrance, CA) column. Mobile phase was 0,1% ammonium hydroxide and 10mM ammonium acetate in water (A) and acetonitrile (B). The gradient was as follows: 0 min 25% B, 9 min 30% B, 16.5 min 50% B, 19.5 min 100% B and 21.5 min 100% B. the flow rate was 0.4 mL/min, the column temperature was set at 27ºC and the injection volume was 2 µL. The mass spectrometer operates in negative electrospray ionisation and data was acquired in Multiple Reaction Monitoring (MRM) mode.

The assignment of bile acid species was performed by direct comparison with commercial standards for CA, β-MCA, CDCA, DCA, HDCA, LCA, UDCA, TCA, TCDCA, TDCA, TLCA, GCA, GCDCA, GDCA and TUDCA, whereas the identification of GLCA, GUDCA, GHDCA, GMCA, TMCA, THDCA and α-MCA is tentative, and their quantification was performed using GCA calibration curve for GLCA, GUDCA, GHDCA and GMCA; TCA calibration curve for TMCA; TUDCA calibration curve for THDCA; and β-MCA calibration curve for α-MCA..

Supplementary material 3b –
SCFA analysis in mouse faeces

Supplementary Table S3. Short-chain fatty acids quantified in faecal samples:

SCFA	GF	EC	ECBSH	Conv	P value
Acetic acid	716.01 ± 330.39[a] (484.52 — 1283.05)	941.20 ± 190.81[a] (696.22 — 1154.17)	734.14 ± 220.02[a] (544.72 — 1112.94)	11277.32 ± 4304.09[b] (7037.61— 17152.56)	<0.001
Propionic acid	41.02 ± 12.65[a] (29.08 — 61.51)	63.27 ± 10.40[a] (51.49 — 73.41)	41.35 ± 14.77[a] (24.55 — 56.69)	2363.06 ± 853.65[b] (1744.59 — 3573.58)	< 0.001
Butyric acid	18.78 ± 20.52 (5.12 — 55.07)	8.15 ± 1.97 (6.36 — 10.96)	9.33 ± 7.81 (4.28 — 23.04)	3037.95 ± 3894.55 (1056.40 — 8879.65)	NS
Isobutyric acid	5.62 ± 2.98 (3.20 — 8.95)	5.18 ± 0.05* (5.15 — 5.22)	ND	71.86 ± 13.65 (40.52 — 100.43)	NA
Valeric acid	8.41 ± 4.03 (5.56 — 11.27)	ND	ND	105.90 ± 123.47 (30.44 — 290.20)	NA
Isovaleric acid	ND	ND	ND	26.63 ± 6.09 (9.18 — 35.19)	NA

Values are expressed as nmol per gram (mean ± SD, and range of quantified metabolites). P values were calculated using ANOVA (considering $P < 0.05$ significant), followed by a Tukey or Dunn's post hoc analysis for the intergroup differences test respectively in those parameters with a significant P value. Different superscript letters indicate statistically significant difference at $P < 0.05$ within each row between the groups. SCFA, Short-chain fatty acids. ND, Not detected (below limit of detection); NA, non-applicable; NS, not significant. * Only detected in two samples

Materials & Methods Analysis of Short Chain Fatty Acids (SCFA)

10 mg of faecal samples were directly weighed in a 1.5 mL LoBind Eppendorf tube and mixed with 10 µL of internal standard mixture (sodium acetate-13C2, propionic acid-d6 and butyric 1,2-13C2 acid) and 990 µL of methanol:water (50:50) mixture. Samples were vortexed for 5 min and centrifuged for 5 minutes at 15000 rpm and 4ºC. A volume of 80 µL of the supernatant was mixed with 10 µL BHA 0.1M and 10 µL EDC 0.25M, vortexed and incubated at RT for 1 hour in darkness to induce acid derivatization. After the incubation, faecal extract was diluted by 20 folds in methanol:water (50:50). Then, 200 µL of diluted sample was extracted by 600 µL of diethyl ether though 10 minutes of vigorous shaking and centrifuged for 5 minutes at 15000 rpm and 4ºC. After the centrifugation, 40 µL of upper organic layer was transferred and evaporated to dryness under a N2 flow. and reconstituted in 200 µL of methanol:water (50:50) for UHPLC-MS/MS analysis.

The quantification of short-chain fatty acids (SCFA) (acetic acid (AA), propionic acid (PA), butyric acid (BA), isobutyric acid (IBA), valeric acid (VA) and isovaleric acid (IVA)) in the same samples described before was performed by ultra-high performance liquid chromatography coupled to triple quadrupole mass spectrometry using an UHPLC 1290 Infinity II Series coupled to a QqQ/MS 6490 Series (Agilent Technologies, Sta. Clara, CA, USA).

The chromatographic separation was performed with a gradient elution on a Kinetex polar C18 (100 x 2.1 mm, 2.6 µm) (Phenomenex, Torrance, CA, USA) column. Mobile phase was 0.1 % formic acid in water with 10 mM of ammonium formate. (A) and 0.1 % formic acid in methanol:2-propanol (9:1; v/v) (B). The gradient was as follows: 0 min 32% B, 4.6 min 60% B, 5.5 min 65% B, 7 min 98% B and 9 min 98% B. the flow rate was 0.3 mL/min, the column temperature was set at 45 ºC and the injection volume was 1 µL. The mass spectrometer operates in positive electrospray ionisation and data was acquired in Multiple Reaction Monitoring (MRM) mode. The assignment and quantification of SCFA was performed by direct comparison with commercial standards
