# Supplementary material for: Microbial bile salt hydrolase activity influences gene expression profiles and gastrointestinal maturation in infant mice
Source: Gut Microbes. 2022 Nov 24;14(1):2149023. doi: 10.1080/19490976.2022.2149023 (PMC9704388; doi:10.1080/19490976.2022.2149023)
Supplement: Supplemental Material [file KGMI_A_2149023_SM9587.zip › Núñez-Sánchez Supp Material 5.pdf]

Supplementary material 5 –
Analysis of GI histology in gnotobiotic mice

a)

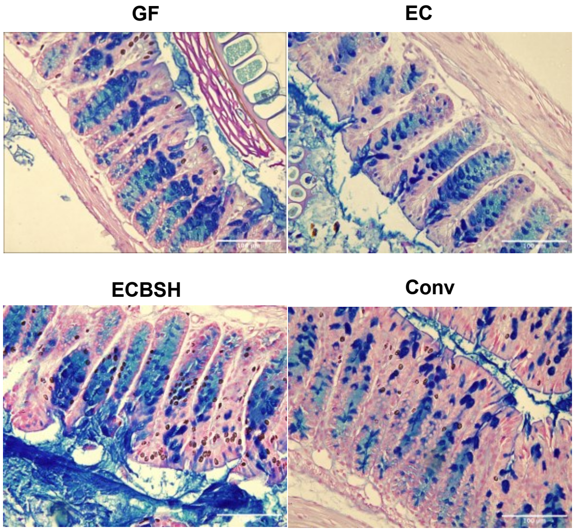

b)

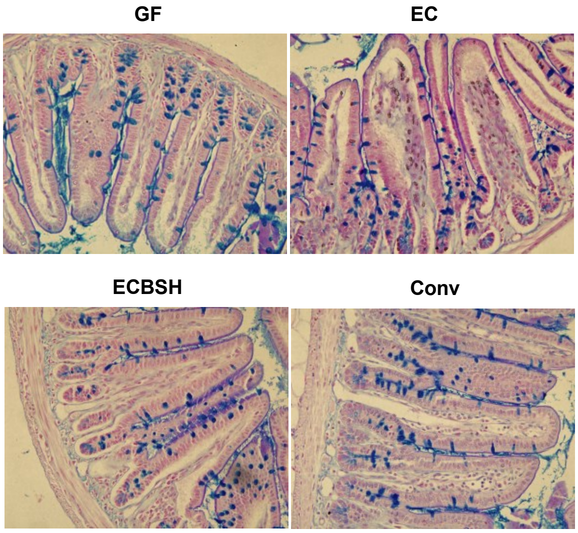

c)

Group	Colon		
	Mucus layer (µm)	No Goblet cells /crypt	No. Goblet cells/ crypt depth (mm)
GF	20.89 ± 1.593	14.91 ± 3.962	123.8 ± 7.566
EC	22.5 ± 2.706	17.72 ± 2.785	131.8 ± 11.99
ECBSH	27.74 ± 2.435	18.51 ± 1.370	121.5 ± 8.606
Conv	36.14 ± 13.51	21.74 ± 5.339	107.5 ± 5.39

d)

Group	Small Intestine			
	Villi height (µm)	Goblet cells/villi	No. Goblet cells/ villi height (mm)	No. Goblet cells/ crypt depth (mm)
GF	202.1 ± 0.303	10.30 ± 3.268	50.99 ± 9.377	92.88 ± 2.674
EC	201.7 ± 8.500	10.32 ± 1.628	51.42 ± 5.725	83.65 ± 15.59
ECBSH	216.6 ± 9.107	10.08 ± 0.250	46.67 ± 1.615	84.96 ± 1.289
Conv	225.4 ± 3.070	9.259 ± 0.121	41.10 ± 0.8369	53.07 ± 4.485

Figure Legend. a) Representative histological images demonstrating colon structure differences between groups. Colon paraffin-embedded sections of 4 µm were stained with Alcian blue and periodic acid-Schiff (PAS) and counterstained with Schiff reagent and Nuclear Fast Red solution (bar 100 µm); **b)** Representative histological images demonstrating SI structure differences between groups. Small intestine paraffin-embedded sections of 4 µm were stained with Alcian blue and periodic acid-Schiff (PAS) and counterstained with Schiff reagent and Nuclear Fast Red solution (bar 100 µm). c) Measurements of various histological parameters in the colon; d) Measurements of various histological parameters in the SI. Statistical analyses were performed using one-way ANOVA and Tukey's multiple comparison test. No statistically significant differences between groups were detected.

Methods. COL and SI samples were collected for histology and immunofluorescence analysis. For histological analysis, half of the samples were stored in Methacarn solution (60 % MeOH: 30 % Chloroform: 10 % acetic acid) for 2 hours at room temperature and dehydrated with 70 % ethanol for 2 h, prior paraffin embedding. Samples for immunohistochemistry were stored in 10 % formalin for 24 h and then dehydrated in 70 % ethanol 24 h prior the embedding process. Paraffin embedding was performed using the histokinette in a 21 h overnight cycle and was blocking was carried on using the console system TissueTek. Paraffin sections of 4 µm were done using the Leica RM2135 rotary Microtome. The analysis of goblet cells crypt depth and villi length was performed as previously described (Las Heras et al., 2019) with some modifications. Paraffin sections of 4 µm were stained with alcian blue and periodic acid-Schiff (PAS) and counterstained with Schiff reagent and Nuclear Fast Red solution. Sections were mounted in DPX mounting reagent (Sigma) and imaged using the Olympus BX51 microscope (Olympus DP71 camera), with a × 40 objective. Image analysis was performed using ImageJ. The sections were blindly scored using a light microscope (Olympus BX51, Olympus, Germany).
