# Supplementary material for: Microbial bile salt hydrolase activity influences gene expression profiles and gastrointestinal maturation in infant mice
Source: Gut Microbes. 2022 Nov 24;14(1):2149023. doi: 10.1080/19490976.2022.2149023 (PMC9704388; doi:10.1080/19490976.2022.2149023)
Supplement: Supplemental Material [file KGMI_A_2149023_SM9587.zip › Núñez-Sánchez Supp Material 6.pdf]

Supplementary material 6 –
Genes and primer sets for transcriptomic analysis in gnotobiotic mice

Supplementary Table S4. Transcriptome analysis gene targets and primer sets

Primers for host transcriptome analysis. Designed in the Assay Design Center from Roche Universal ProbeLibrary (version 2.52, 2016).

Gene Symbol	Official full name	Gene ID	mRNA Accession	Forward	Reverse	Probe
Actb	Actin, beta	11461	NM_007393	aaggccaaccgtgaaaagat	gtggtacgaccagaggcatac	#56
Lgr5	leucine rich repeat containing G protein coupled receptor 5	14160	NM_010195.2	gactttaactggagcaaagatct ca	cgagtaggttgtaagacaaatct agc	#60
Sox9	SRY (Sex determining region Y)-box 9	20682	NM_011448.4	gtacccgcatctgcacaac	ctcctccacgaagggtctct	#66
Ascl2	Achaete-scute family bHLH transcription factor 2	17173	NM_008554.3	gagagctaagcccgatgga	aggtccaccaggagtcacc	#17
Notch2	Notch 2	18129	NM_010928.2	ccatttcaagtgttcgtgtcc	cacattcatcgatgttctcttca	#40
Hes1	Hes family bHLH transcription facto 1	15205	NM_008235.2	tgccagctgatataatggagaa	ccatgataggctttgatgacttt	#20
Prmd1	PR domain containing 1, with ZNF domain	12142	NM_007548.4	acgtgtgggtacgaccttg	ccatgtccattttcatgatcc	#53
Mki67	Antigen identified by monoclonal antibody Ki 67	17345	NM_001081117.2	agggtaactcgtggaaccaa	tcttaacttcttggtgcatacaat g	#88
Vim	Vimentin	22352	NM_011701.4	ccaaccttttcttccctgaac	ttgagtgggtgtcaaccaga	#109
Egfr	Epidermal growth factor receptor	13649	NM_007912.4	gccacgccaactgtacctat	gccacacttcacatccttga	#107
Klf4	Kruppel-like factor 4	16600	NM_010637.3	cgggaagggagaagacact	gagttcctcacgccaacg	#62
Mmp2	Matrix metallopeptidase 2	17390	NM_008610.3	gtgggacaagaaccagatcac	gcatcatccacggtttcag	#85
Mmp9	Matriz metallopeptidase 9	17395	NM_013599.4	agacgacatagacggcatcc	tcggctgtggttcagttgt	#19
Ocln	Occludin	18260	NM_008756.2	gtccgtgaggccttttga	ggtgcataatgattgggtttg	#10
Tjp1	Tight junction protein 1	21872	NM_009386.2	tgcagacccagcaaaggt	ggttttgtctcatcatttcttcag	#12
Tjp2	Tight Junction protein 2	21873	NM_001198985.1	catcagcgcacacagagc	gtccctggacacaaagtcg	#1
Muc2	Mucin 2	17831	NM_023566.3	acctccaggttcaacaccag	gttggccctgttgtggtct	#10
Alpi	Alkaline phosphatase, intestinal	24197	NM_001081082.2	aaacgtggctgtcaaagcat	tcaaagaggcccatgaggt	#3
Vil1	Villin 1	22349	NM_009509.2	gatctccctgagggtgtgg	agtgaagtcttcggtggacag	#3
Chga	Chromogranin A	12652	NM_007693.2	cgatccagaaagatgatggtc	cggaagcctctgtctttcc	#58
Cdkn1a	Cyclin-dependent kinase inhibitor 1A (P21)	12575	NM_007669.5	tccacagcgatatccagaca	ggacatcaccaggattggac	#21
Reg3g	Regenerating islet-derived 3 gamma	19695	NM_011260.2	accatcaccatcatgtcctg	ggcatctttcttggcaactt	#108
Il10	Interleukin 10	16153	NM_010548	cagagccacatgctcctaga	tgtccagctggtcctttgtt	#41
Il17a	Interleukin 17A	16171	NM_010552.3	cagggagagcttcatctgtgt	gctgagctttgagggatgat	#74
Ifnγ	Interferon gamma	15978	NM_008337	atctggaggaactggcaaaa	ttcaagacttcaaagagtctgag g	#21
Fgf15	Fibroblast growth factor 15	14170	NM_008003.2	ggcaagatatacgggctgat	tccatttcctccctgaaggt	#69
Nr1h4	Nuclear receptor subfamily 1, group H, member 4	20186	NM_009108.2	gaaaatccaattcagattagtctt cac	ccgcgtgttctgttagcat	#83
Gpbar1	G protein-couple bile acid receptor 1	227289	NM_174985.1	gctagggctctcacctgga	ccccaacacagcaagaagag	#51
Cyp7a1	Cytochrome P450, family 7, subfamily a, polypeptide 1	13122	NM_007824.2	ggagcttatttcaaatgatcagg	ttggccagcactctgtaatg	#110
Abcg5	ATP binding cassette subfamily G member 5	27409	NM_031884.2	tcctgcatgtgtcctacagc	atttgcctgtcccacttctg	#31
Abcg8	ATP binding cassette subfamily G member 8	67470	NM_026180.3	aaccctgcggacttctacg	ctgcaagagactgtgccttct	#10
