# Supplementary material for: Microbial bile salt hydrolase activity influences gene expression profiles and gastrointestinal maturation in infant mice
Source: Gut Microbes. 2022 Nov 24;14(1):2149023. doi: 10.1080/19490976.2022.2149023 (PMC9704388; doi:10.1080/19490976.2022.2149023)
Supplement: Supplemental Material [file KGMI_A_2149023_SM9587.zip › Núñez-Sánchez Supp Material 7.pdf]

Supplementary material 7 –
Analysis of Colon organoids controls for viability, proliferation, apoptosis and measures of organoid size

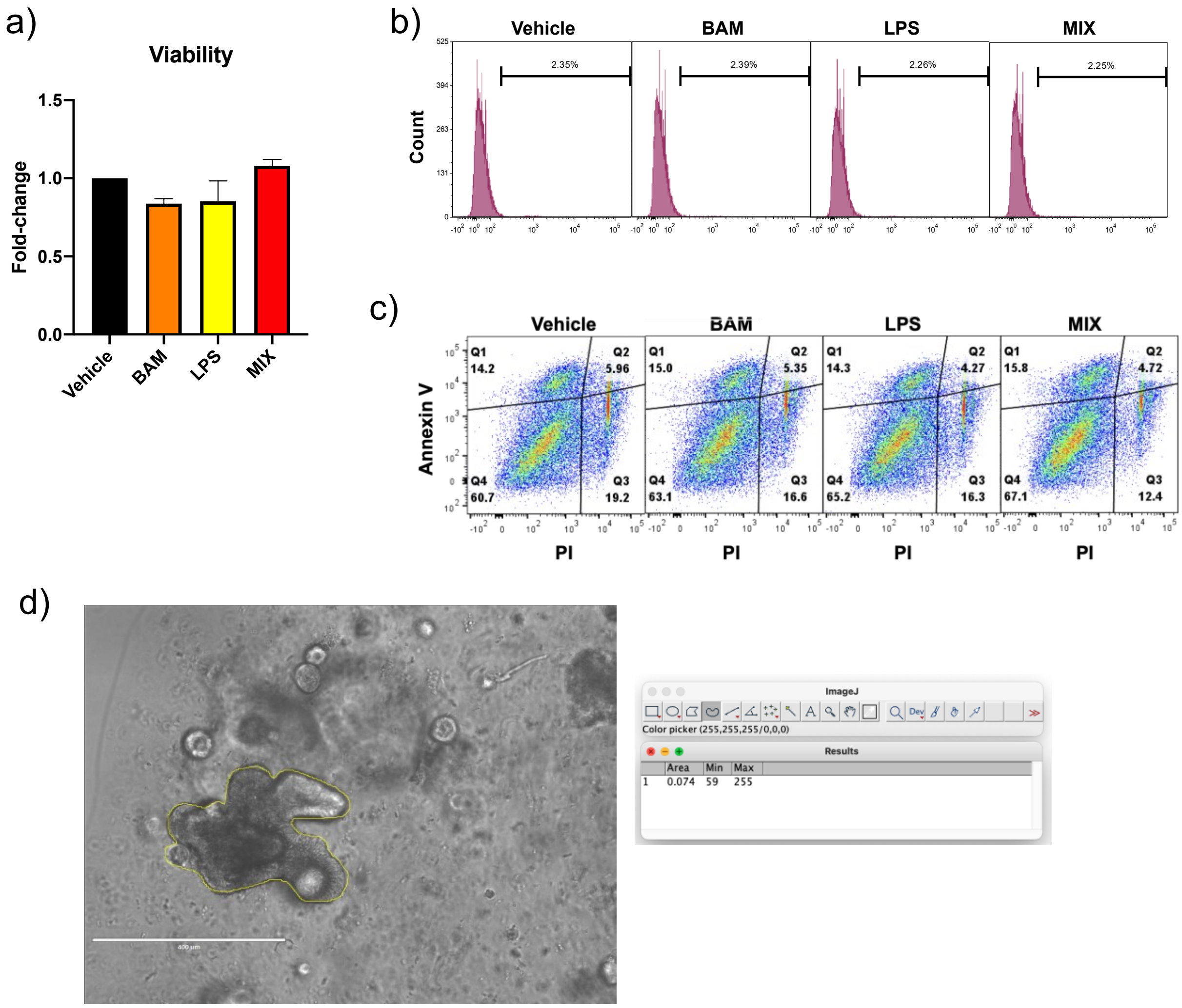

Figure Legend. a) Viability test of COLOrg exposed to different treatments. Values are represented as the fold-change between the treatments and the vehicle; b) Proliferation difference between the treatments after 7 days incubation; c) Difference in apoptosis by Annexin V/PI represented as the percentage of death cells; d) Representative image demonstrating organoid size measurements. Images from ColORG were taken by live transmission light microscopy imaging using the EVOS FL inverted microscope (Invitrogen) at 10X and 20X magnification. Acquired digital images were processed in ImageJ. ColORG were counted and surface area was calculated by outlining manually the area of ColORG. Values from each well were averaged and graphed in Figure 3.

Methods:

Crypt isolation, organoid development & treatment. Once crypts were isolated they were resuspended in the pertinent amount of Cultrex Basement Membrane Extract (BME) (R&D Systems). Twenty microliters of BME containing the crypts were seeded in 48-well plates for RT-qPCR analyses and 8 μL in 96-well plates for viability and microscopy analysis. Plates were incubated inverted at 37ºC for 30 min to allow the matrix to solidify and then conditioned L-WRN media (see Supplementary Methods) was added containing L-WRN media:Advanced DMEM/F12 1:1 supplemented with 1X Glutamax, 1% Penicillin/Streptomycin, 10 mM HEPES, 1X N2 supplement, 1X B27 supplement, 1mM N-Acetylcysteine, 50 ng/mL recombinant murine EGF, 10 mM nicotinamide, 10 mM Y-27632, 500 nM A-83-01, 10 μM SB202190 and 5 μM CHIR-99021. After 24 h media was change into mCOL media containing L-WRN media:Advanced DMEM/F12 1:1 supplemented with 1X Glutamax, 1% Penicillin/Streptomycin, 10 mM HEPES, 1X N2 supplement, 1X B27 supplement, 1mM N-Acetylcysteine, 50 ng/mL recombinant murine EGF and 10 μM Y-27632 to allow crypts for differentiation and treated with 10 mM bile acid mix (BAM; 8 μM CA, 1,5 μM UDCA, 0,5 μM CDCA), 100 ng/mL lipopolysaccharide (LPS) or LPS+BAM (MIX). Control wells with the same volume of DMSO were included. COL organoids were incubated for 7 days with changes of media every second day.After 7 days of incubation, organoid viability was evaluated by measuring the reduction of measured MTT [3-(4,5- dimethylthiazol-2-yl)-2,5-diphenyltetrazolium bromide] to formazan as described by Grabinger et al (2014) with some modifications. Cell proliferation was evaluated by flow cytometry using the Click-iT EdU Proliferation Kit (Invitrogen) as specified by the manufacturer. Apoptosis was evaluated by flow cytometry using the Annexin V/PI assay using APC-Annexin V and PI from Biolegend following manufacturer recommendations.

Reference

Grabinger T, Luks L, Kostadinova F, Zimberlin C, Medema JP, Leist M, et al. Ex vivo culture of intestinal crypt organoids as a model system for assessing cell death induction in intestinal epithelial cells and enteropathy. *Cell Death Dis* 2014; 5:e1228.
