# Supplementary material for: Microbial bile salt hydrolase activity influences gene expression profiles and gastrointestinal maturation in infant mice
Source: Gut Microbes. 2022 Nov 24;14(1):2149023. doi: 10.1080/19490976.2022.2149023 (PMC9704388; doi:10.1080/19490976.2022.2149023)

Supplementary material 1 –
in silico analysis of BSH in human datasets

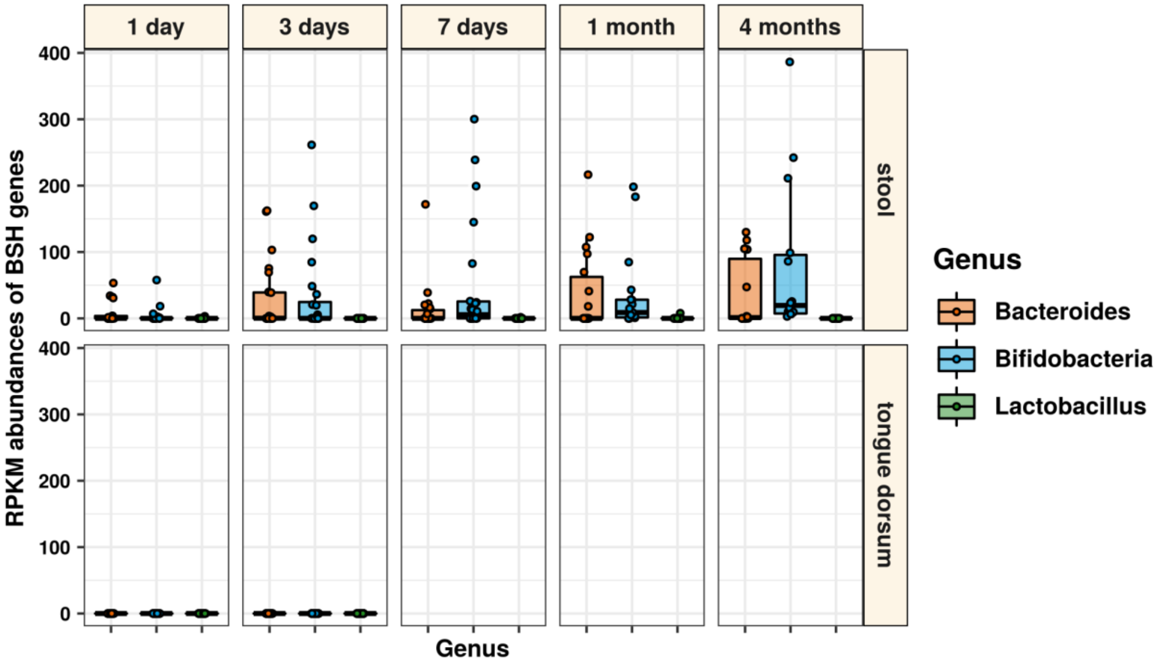

Quantification of bile salt hydrolases (BSH) in an infant metagenomic cohort
ShortBRED (v0.9.4) was used to quantify composition and abundance of BSH genes (*Kaminski et al*., 2015). We mainly focused on a total of 31 BSH protein sequences that belong to different species from *Lactobacillus* (n=11), *Bacteroides* (n=10), and *Bifidobacteria* (n=10) genus (Table S1). ShortBRED specific unique marker sequences were generated from selected BSH protein sequences with following parameter (--clustid 0.85 --ref Uniref90.fasta) and subsequently used to quantify BSH genes in metagenomic samples using default parameters. We specifically focused on an infant metagenomic cohort from *Ferretti et al*. (2018) for which multiple infant samples (stool and tongue dorsum) were collected in the first four months of life. Briefly, raw fastq files were downloaded using the fastq-dump utility script in SRA Toolkit. Quality filtering and host decontamination of raw sequencing data was carried out using Kneaddata tool with default parameters. We processed 136 valid SRA runs (from Infants) associated with the NCBI Bio project PRJNA352475 that consisted of an average of 20.2 million quality filtered reads. Individual marker level abundance in reads per kilobase per million reads (RPKM) was further grouped by its genus level assignment.

References

Ferretti P, Pasolli E, Tett A, Asnicar F, Gorfer V, Fedi S, et al. Mother-to-Infant Microbial Transmission from Different Body Sites Shapes the Developing Infant Gut Microbiome. Cell Host Microbe 2018; 24:133-45 e5.
Kaminski J, Gibson MK, Franzosa EA, Segata N, Dantas G, Huttenhower C: High-Specificity Targeted Functional Profiling in Microbial Communities with ShortBRED. PLoS Comput Biol 2015, 11:e1004557.

Supplementary Material 1 cont…

Supplementary Table S1. Metagenomic query sequences used in previous figure.

NCBI accessions	Genus	Species/ Strains
WP_003546965.1	Lactobacillus	Lactobacillus acidophilus NCFM
WP_010690294.1	Lactobacillus	Lactobacillus animalis
AEB72500.1	Lactobacillus	Lactobacillus buchneri NRRL B-30929
AEZ06356.1	Lactobacillus	Lactobacillus fermentum NCDO 394
ABJ59469.1	Lactobacillus	Lactobacillus gasseri ATC 33323
AAS09178.1	Lactobacillus	Lactobacillus johnsonii NCC533
ADN97280.1	Lactobacillus	Lactobacillus plantarum ST-III
EDX41535.1	Lactobacillus	Lactobacillus reuteri 100-23
ACL98204.1	Lactobacillus	Lactobacillus salivarius JCM1046
WP_056974571.1	Lactobacillus	Lactobacillus vaginalis
CCC80500.1	Lactobacillus	Lactobacillus plantarum WCFS1
WP_195394019.1	Bifidobacteria	Bifidobacteria adolescentis
WP_045919813.1	Bifidobacteria	Bifidobacteria angulatum
WP_130079430.1	Bifidobacteria	Bifidobacteria animalis
WP_053824989.1	Bifidobacteria	Bifidobacteria bifidum
AUD87005.1	Bifidobacteria	Bifidobacteria breve
WP_039198334.1	Bifidobacteria	Bifidobacteria catenulatum
WP_006294150.1	Bifidobacteria	Bifidobacteria gallicum
WP_032743741.1	Bifidobacteria	Bifidobacteria longum
WP_209107517.1	Bifidobacteria	Bifidobacteria pseudocatenulatum
WP_026646013.1	Bifidobacteria	Bifidobacteria ruminatium
EDU99086.1	Bacteroides	Bacteroides coprocola DSM 17136
EEO44970.1	Bacteroides	Bacteroides dorei 5_1_36/D4
EEC53612.1	Bacteroides	Bacteroides eggerthii DSM 20697
BAD50536.1	Bacteroides	Bacteroides fragilis YCH46
EDV03500.1	Bacteroides	Bacteroides intestinalis DSM 17393
EDO11390.1	Bacteroides	Bacteroides ovatus ATCC8483
AAO77193.1	Bacteroides	Bacteroides thetaoitaomicron VPI_5482
EDO55730.1	Bacteroides	Bacteroides uniformis ATCC 8492
ABR41596.1	Bacteroides	Bacteroides vulgatus ATCC 8482
CDM05097.1	Bacteroides	Bacteroides xylamisolvens SD CC 1b

Supplementary material 2
Construction of *E. coli* BSH

a)

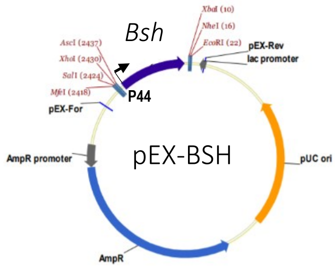

b)

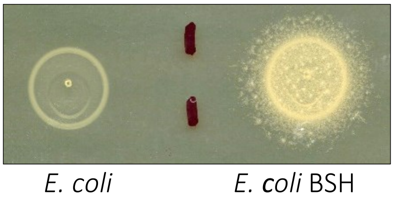

E. coli BSH Strain construction a) To construct an *Escherichia coli* strain expressing bile salt hydrolase (BSH) using a multi-copy plasmid and strong promoter, the sequence corresponding to the *L. salivarius* JCM1046 BSH1 (accession number FJ591081.1) was synthesised downstream of the P44 promoter into the standard cloning vector pEX-A128 (Eurofins Genomics) to generate pEX-BSH. The plasmid was then purified and individually transformed into the commensal *E. coli* MG1655 (courtesy of the Yale Culture Collection). b) 5μl of overnight cultures were plated onto LB agar plates containing 5mM TDCA and incubated for 48 hours at 37° C.

Supplementary material 3a –
Bile acid analysis in mouse faeces

Supplementary Table S2. Bile acids quantified in faecal samples:

Bile Acids	GF	EC	ECBSH	Conv	P value
Total BA	2343.47 ± 1186.83<br>(787.80 – 3491.37)	4174.40 ± 1494.83<br>(1811.51 – 5572.16)	3402.72 ± 2930.61<br>(651.71 – 7029.69)	4387.48 ± 2725.18<br>(709.87 – 7036.98)	NS
Unconjugated BA	24.91 ± 6.44 ^{a}<br>(14.96 – 29.73)	33.27 ± 7.13 ^{a}<br>(25.23 – 43.44)	363.34 ± 232.81 ^{a}<br>(119.43 – 601.38)	4266.61 ± 2705.08 ^{b}<br>(692.06 – 6944.07)	< 0.001
Conjugated BA	2318.57 ± 1188.41^{a,b}<br>(759.44 – 3469.54)	4141.13 ± 1489.97^{a}<br>(1786.28 – 5528.72)	3039.37 ± 2704.53 ^{a,b}<br>(532.28 – 6428.31)	120.87 ± 50.39 ^{b}<br>(80.81 – 193.08)	0.022
Primary BA	2342.96 ± 1186.79<br>(787.37 – 3490.86)	4173.85 ± 1494.77<br>(1811.12 – 5571.47)	3401.87 ± 2930.64<br>(651.33 – 7028.52)	3364.95 ± 2075.66<br>(671.00 – 5467.13)	NS
Unconjugated	24.49 ± 6.35^{a}<br>(14.66 – 29.20)	32.90 ± 7.03^{a}<br>(24.96 – 42.98)	362.56 ± 232.94^{a}<br>(119.07 – 600.21)	3253.75 ± 2059.11 ^{b}<br>(593.57 – 5378.20)	< 0.001
CA	10.99 ± 0.16^{a}<br>(10.81 – 11.13)	13.29 ± 2.26^{a,b}<br>(10.68 – 16.04)	198.96 ± 128.40^{b}<br>(66.39 – 325.5)	169.07 ± 197.71^{a,b}<br>(35.19 – 459.30)	< 0.001
CDCA	0.11 ± 0.02^{a}<br>(0.08 – 0.13)	0.23 ± 0.08^{a,b}<br>(0.18 – 0.36)	6.96 ± 5.23^{b}<br>(1.68 – 12.81)	12.37 ± 14.11^{b}<br>(1.35 – 32.94)	< 0.001
α-MCA	6.02 ± 0.91^{a}<br>(4.73 – 6.74)	5.68 ± 1.21^{a}<br>(4.25 – 6.69)	7.10 ± 1.95^{a}<br>(4.31 – 9.24)	1801.73 ± 1158.03^{b}<br>(445.3 – 3254)	< 0.001
β-MCA	11.36 ± 2.00^{a}<br>(9.43 – 14.30)	13.21 ± 3.75^{a}<br>(9.44 – 19.34)	143.69 ± 96.05^{a,b}<br>(42.01 – 244.2)	1248.94 ± 855.00^{b}<br>(107.6 – 2026)	0.003
UDCA	0.43 ± 0.03^{a}<br>(0.37 – 0.45)	0.49 ± 0.10^{a}<br>(0.37 – 0.62)	5.84 ± 3.91^{a,b}<br>(1.75 – 10.13)	21.64 ± 15.58^{b}<br>(4.15 – 39.34)	0.003
Conjugated	2318.46 ± 1188.36^{a,b}<br>(759.40 – 3469.37)	4140.95 ± 1489.92^{a}<br>(1786.16 – 5528.49)	3039.31 ± 2704.53^{a,b}<br>(532.26 – 6428.31)	111.21 ± 41.00^{b}<br>(77.43 – 169.53)	0.022
TCA	683.97 ± 361.91^{a,b}<br>(222.9 – 1030)	1309.19 ± 514.81^{a}<br>(526.3 – 1732)	925.05 ± 831.43^{a,b}<br>(171.1 – 1952)	40.05 ± 14.37^{b}<br>(28.22 – 58.23)	0.022
TCDCA	30.84 ± 17.74^{a}<br>(10.53 – 53.38)	76.44 ± 10.75^{b}<br>(67.55 – 90.35)	33.11 ± 35.11^{a}<br>(3.74 – 87.29)	4.39 ± 2.54^{a}<br>(2.57 – 8.14)	0.003
TMCA	1568.88 ± 790.41^{a,b}<br>(517.8 – 2339)	2704.88 ± 948.39^{a}<br>(1238 – 3786)	2041.08 ± 1800.48^{a,b}<br>(352.4 – 4289)	63.63 ± 23.62^{b}<br>(44.34 – 98.04)	0.023
TUDCA	33.64 ± 20.36^{a,b}<br>(7.74 – 54.95)	64.14 ± 29.02^{a}<br>(21.08 – 100.4)	38.83 ± 40.38^{a,b}<br>(4.60 – 97.29)	2.17 ± 0.97^{b}<br>(1.28 – 3.37)	0.035
GCA	0.12 ± 0.06<br>(0.06 – 0.20)	0.23 ± 0.09<br>(0.10 – 0.35)	0.19 ± 0.15<br>(0.05 – 0.38)	0.14 ± 0.09<br>(0.06 – 0.28)	NS
GCDCA	ND	0.01 ± 0.00<br>(0.01 – 0.01)	0.01 ± 0.01<br>(0.00 – 0.01)	0.01 ± 0.01<br>(0.00 – 0.02)	NA
GMCA	0.89 ± 0.41<br>(0.29 – 1.37)	1.35 ± 0.43<br>(0.70 – 1.84)	1.02 ± 0.85<br>(0.25 – 2.31)	0.82 ± 0.61<br>(0.08 – 1.44)	NS
GUDCA	0.01 ± 0.01<br>(0.00 – 0.02)	0.01 ± 0.00<br>(0.01 – 0.01)	0.01 ± 0.01<br>(0.00 – 0.03)	0.01 ± 0.01<br>(0.00 – 0.02)	NS
Secondary BA	0.51 ± 0.09^{a}<br>(0.41 – 0.60)	0.55 ± 0.14^{a}<br>(0.39 – 0.71)	0.85 ± 0.62^{a}<br>(0.32 – 1.78)	1350.41 ± 195.09^{b}<br>(1196.55 – 1569.85)	< 0.001
Unconjugated	0.40 ± 0.09^{a}<br>(0.30 – 0.53)	0.37 ± 0.14^{a}<br>(0.21 – 0.56)	0.78 ± 0.64^{a}<br>(0.20 – 1.73)	1338.65 ± 200.65^{b}<br>(1188.81 – 1565.87)	< 0.001
DCA	0.34 ± 0.09^{a}<br>(0.29 – 0.51)	0.35 ± 0.14^{a}<br>(0.19 – 0.54)	0.75 ± 0.63^{a}<br>(0.19 – 1.67)	1243.50 ± 168.12^{b}<br>(1117 – 1434)	< 0.001
LCA	0.02 ± 0.01^{a}<br>(0.01 – 0.02)	0.02 ± 0.00^{a,b}<br>(0.02 – 0.02)	0.03 ± 0.02^{a,b}<br>(0.01 – 0.06)	32.16 ± 5.15^{b}<br>(26.89 – 39.01)	0.013
HDCA	ND	ND	ND	48.08 ± 34.81<br>(8.60 – 92.67)	NA
Conjugated	0.11 ± 0.05^{a}<br>(0.04 – 0.17)	0.18 ± 0.07^{a}<br>(0.12 – 0.27)	0.08 ± 0.05^{a}<br>(0.02 – 0.12)	11.75 ± 10.38^{b}<br>(3.98 – 23.55)	0.003
TDCA	ND	ND	ND	6.79 ± 7.21<br>(2.27 – 17.44)	NA
TLCA	0.11 ± 0.05<br>(0.04 – 0.17)	0.18 ± 0.07<br>(0.12 – 0.27)	0.08 ± 0.05<br>(0.02 – 0.12)	0.44 ± 0.50<br>(0.07 – 1.15)	NS
THDCA	ND	ND	ND	2.24 ± 1.73<br>(0.99 – 4.70)	NA
GDCA	ND	ND	ND	0.16 ± 0.13<br>(0.03 – 0.31)	NA
GLCA	ND	ND	ND	0.02 ± 0.01<br>(0.01 – 0.02)	NA
GHDCA	ND	ND	ND	0.02 ± 0.01<br>(0.01 – 0.03)	NA

Legend on next page

Legend Table S2: Values are expressed as nmol per gram (mean ± SD, and range of quantified metabolites). *P values* were calculated using ANOVA test in those parameters with a normal distribution (Total BA, Unconjugated BA, Conjugated BA, Primary BA, Unconjugated primary BA, Conjugated primary BA, Secondary BA, Unconjugated secondary BA, Conjugated secondary BA, DCA, α-MCA, TCA, TCDCA, TLCA, GCA, TUDCA, TMCA and GMCA), whereas *P* values for parameters without a normal distribution (CA, CDCA, LCA, UDCA, β-MCA, and GUDCA) were calculated using a Kruskal-Wallis test (considering $P < 0.05$ significant), followed by a Tukey or Dunn's post hoc analysis for the intergroup differences test respectively in those parameters with a significant *P value*. Different superscript letters indicate statistically significant difference at $P < 0.05$ within each row between the groups. BA, Bile acids; CA, cholic acid; CDCA, chenodeoxycholic acid; α-MCA, α-muricholic acid; β-MCA, β-muricholic acid; TCA, taurocholic acid; TCDCA, taurochenodeoxycholic acid; TMCA, tauromuricholic acid; GCA, glycocholic acid; GCDCA, glycochenodeoxycholic acid; GMCA, glycomuricholic acid; DCA, deoxycholic acid; LCA, lithocholic acid; UDCA, ursodeoxycholic acid; HDCA, hyodeoxycholic acid; TDCA, taurodeoxycholic acid; TLCA, taurolithocholic acid; TUDCA, tauroursodeoxycholic acid; THDCA, taurohyodeoxycholic acid; GDCA, glycodeodeoxycholic acid; GLCA, glycolithocholic acid; GUDCA, glycoursodeoxycholic acid; GHDCA, glycohyodeoxycholic acid; ND, Non detected (below limit of detection); NA, non-applicable; NS, not significant.

Supplementary material 3a – *Bile acid analysis in mouse faeces*

Materials and Methods Bile acid analysis

Samples (10 mg faecal matter) were placed in a 2 mL Eppendorf tube and mixed with 20 µL of internal standard solution (Cholic acid-d4 and Taurocholic Acid-d5), 800 µL of 0.1M NaOH and a steel bead. Samples were homogenized with a bullet blender for 3 minutes at speed 8 and vortexed for 5 minutes and incubated at 60 ºC for 1 hour. Afterwards, a volume of 600 µL of water was added and the samples were centrifuged for 10 minutes at 15000 rpm and 4ºC. Supernatants were loaded to a SPE cartridge (Oasis HLB 30 mg sorbent) previously conditioned with 1 mL of methanol and 1 mL of water. Cartridges were washed with 1 mL of water, 1 mL of hexane and 1 mL of water. Then, cartridges were dried under high vacuum and compounds were eluted with 500 µL of methanol twice. The elutes were evaporated to dryness in a SpeedVac at 45 ºC. and reconstituted with 100 µL of methanol for their analysis by UHPLC-MS/MS.

The quantification of 15 bile acids (see Supplementary Methods) in 80 mouse faecal samples were performed by ultra-high performance liquid chromatography coupled to triple quadrupole mass spectrometry using an UHPLC 1290 Infinity II Series coupled to a QqQ/MS 6490 Series (Agilent Technologies, Sta. Clara, CA, USA).

The chromatographic separation was performed with a gradient elution on a Kinetex EVO C18 (150 x 2.1 mm, 2.6 µm) (Phenomenex, Torrance, CA) column. Mobile phase was 0,1% ammonium hydroxide and 10mM ammonium acetate in water (A) and acetonitrile (B). The gradient was as follows: 0 min 25% B, 9 min 30% B, 16.5 min 50% B, 19.5 min 100% B and 21.5 min 100% B. the flow rate was 0.4 mL/min, the column temperature was set at 27ºC and the injection volume was 2 µL. The mass spectrometer operates in negative electrospray ionisation and data was acquired in Multiple Reaction Monitoring (MRM) mode.

The assignment of bile acid species was performed by direct comparison with commercial standards for CA, β-MCA, CDCA, DCA, HDCA, LCA, UDCA, TCA, TCDCA, TDCA, TLCA, GCA, GCDCA, GDCA and TUDCA, whereas the identification of GLCA, GUDCA, GHDCA, GMCA, TMCA, THDCA and α-MCA is tentative, and their quantification was performed using GCA calibration curve for GLCA, GUDCA, GHDCA and GMCA; TCA calibration curve for TMCA; TUDCA calibration curve for THDCA; and β-MCA calibration curve for α-MCA..

Supplementary material 3b –
SCFA analysis in mouse faeces

Supplementary Table S3. Short-chain fatty acids quantified in faecal samples:

SCFA	GF	EC	ECBSH	Conv	P value
Acetic acid	716.01 ± 330.39[a] (484.52 — 1283.05)	941.20 ± 190.81[a] (696.22 — 1154.17)	734.14 ± 220.02[a] (544.72 — 1112.94)	11277.32 ± 4304.09[b] (7037.61— 17152.56)	<0.001
Propionic acid	41.02 ± 12.65[a] (29.08 — 61.51)	63.27 ± 10.40[a] (51.49 — 73.41)	41.35 ± 14.77[a] (24.55 — 56.69)	2363.06 ± 853.65[b] (1744.59 — 3573.58)	< 0.001
Butyric acid	18.78 ± 20.52 (5.12 — 55.07)	8.15 ± 1.97 (6.36 — 10.96)	9.33 ± 7.81 (4.28 — 23.04)	3037.95 ± 3894.55 (1056.40 — 8879.65)	NS
Isobutyric acid	5.62 ± 2.98 (3.20 — 8.95)	5.18 ± 0.05* (5.15 — 5.22)	ND	71.86 ± 13.65 (40.52 — 100.43)	NA
Valeric acid	8.41 ± 4.03 (5.56 — 11.27)	ND	ND	105.90 ± 123.47 (30.44 — 290.20)	NA
Isovaleric acid	ND	ND	ND	26.63 ± 6.09 (9.18 — 35.19)	NA

Values are expressed as nmol per gram (mean ± SD, and range of quantified metabolites). P values were calculated using ANOVA (considering $P < 0.05$ significant), followed by a Tukey or Dunn's post hoc analysis for the intergroup differences test respectively in those parameters with a significant P value. Different superscript letters indicate statistically significant difference at $P < 0.05$ within each row between the groups. SCFA, Short-chain fatty acids. ND, Not detected (below limit of detection); NA, non-applicable; NS, not significant. * Only detected in two samples

Materials & Methods Analysis of Short Chain Fatty Acids (SCFA)

10 mg of faecal samples were directly weighed in a 1.5 mL LoBind Eppendorf tube and mixed with 10 µL of internal standard mixture (sodium acetate-13C2, propionic acid-d6 and butyric 1,2-13C2 acid) and 990 µL of methanol:water (50:50) mixture. Samples were vortexed for 5 min and centrifuged for 5 minutes at 15000 rpm and 4ºC. A volume of 80 µL of the supernatant was mixed with 10 µL BHA 0.1M and 10 µL EDC 0.25M, vortexed and incubated at RT for 1 hour in darkness to induce acid derivatization. After the incubation, faecal extract was diluted by 20 folds in methanol:water (50:50). Then, 200 µL of diluted sample was extracted by 600 µL of diethyl ether though 10 minutes of vigorous shaking and centrifuged for 5 minutes at 15000 rpm and 4ºC. After the centrifugation, 40 µL of upper organic layer was transferred and evaporated to dryness under a N2 flow. and reconstituted in 200 µL of methanol:water (50:50) for UHPLC-MS/MS analysis.

The quantification of short-chain fatty acids (SCFA) (acetic acid (AA), propionic acid (PA), butyric acid (BA), isobutyric acid (IBA), valeric acid (VA) and isovaleric acid (IVA)) in the same samples described before was performed by ultra-high performance liquid chromatography coupled to triple quadrupole mass spectrometry using an UHPLC 1290 Infinity II Series coupled to a QqQ/MS 6490 Series (Agilent Technologies, Sta. Clara, CA, USA).

The chromatographic separation was performed with a gradient elution on a Kinetex polar C18 (100 x 2.1 mm, 2.6 µm) (Phenomenex, Torrance, CA, USA) column. Mobile phase was 0.1 % formic acid in water with 10 mM of ammonium formate. (A) and 0.1 % formic acid in methanol:2-propanol (9:1; v/v) (B). The gradient was as follows: 0 min 32% B, 4.6 min 60% B, 5.5 min 65% B, 7 min 98% B and 9 min 98% B. the flow rate was 0.3 mL/min, the column temperature was set at 45 ºC and the injection volume was 1 µL. The mass spectrometer operates in positive electrospray ionisation and data was acquired in Multiple Reaction Monitoring (MRM) mode. The assignment and quantification of SCFA was performed by direct comparison with commercial standards

Supplementary Material 4

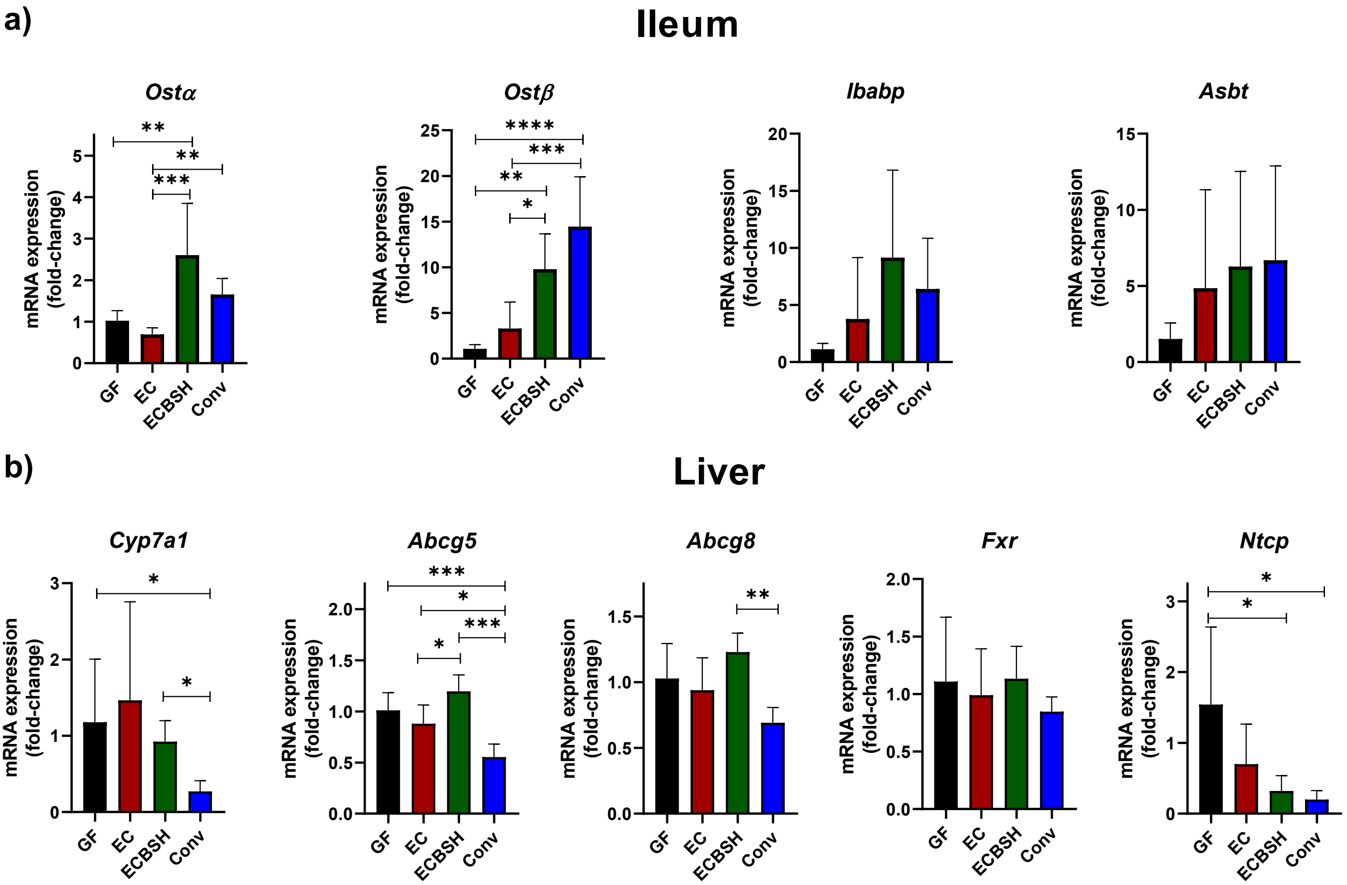

Figure S4. Bacterial BSH influences BA transporters genes expression. a) Terminal ileum; and b) liver gene expression by RT-qPCR. All values are represented as the fold change of relative mRNA expression between the condition and the GF group (mean ± SD). Statistical analyses were conducted using ANOVA test in those parameters with a normal distribution (*Ostβ*, *Abcg5, Fxr,* and *Ntcp*), whereas P values for parameters without a normal distribution (*Ibabp, Asbt, Ostα, Cyp7a1* and *Abcg8*) were calculated using a Kruskal-Wallis test (considering P < 0.05 significant), followed by a Tukey or Dunn's post hoc analysis for the intergroup differences test respectively in those parameters with a significant P value. * p < 0.05; ** p < 0.01; *** p < 0.001; **** p < 0.00001.

Supplementary material 5 –
Analysis of GI histology in gnotobiotic mice

a)

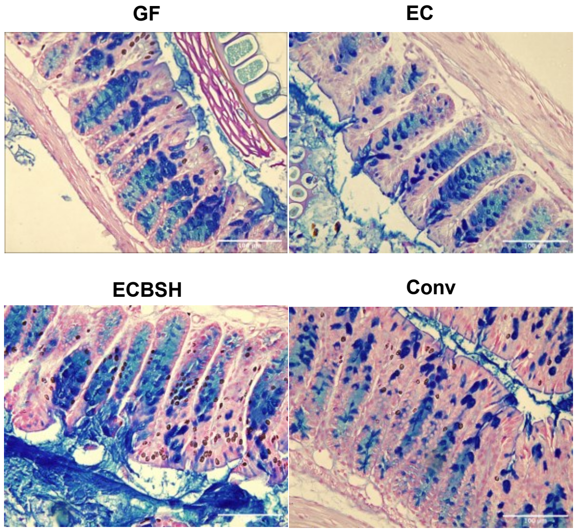

b)

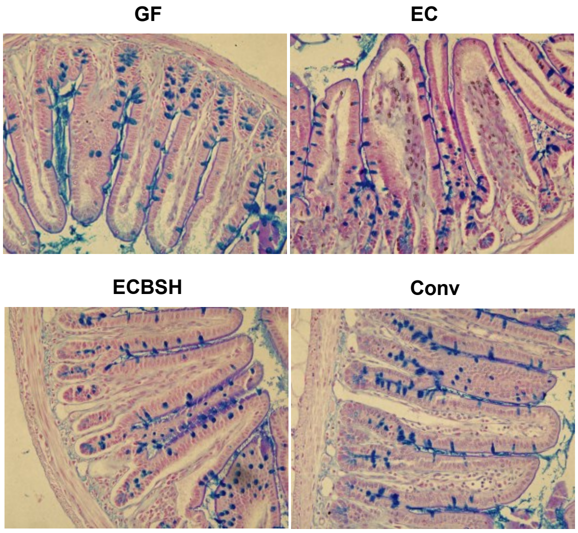

c)

Group	Colon		
	Mucus layer (µm)	No Goblet cells /crypt	No. Goblet cells/ crypt depth (mm)
GF	20.89 ± 1.593	14.91 ± 3.962	123.8 ± 7.566
EC	22.5 ± 2.706	17.72 ± 2.785	131.8 ± 11.99
ECBSH	27.74 ± 2.435	18.51 ± 1.370	121.5 ± 8.606
Conv	36.14 ± 13.51	21.74 ± 5.339	107.5 ± 5.39

d)

Group	Small Intestine			
	Villi height (µm)	Goblet cells/villi	No. Goblet cells/ villi height (mm)	No. Goblet cells/ crypt depth (mm)
GF	202.1 ± 0.303	10.30 ± 3.268	50.99 ± 9.377	92.88 ± 2.674
EC	201.7 ± 8.500	10.32 ± 1.628	51.42 ± 5.725	83.65 ± 15.59
ECBSH	216.6 ± 9.107	10.08 ± 0.250	46.67 ± 1.615	84.96 ± 1.289
Conv	225.4 ± 3.070	9.259 ± 0.121	41.10 ± 0.8369	53.07 ± 4.485

Figure Legend. a) Representative histological images demonstrating colon structure differences between groups. Colon paraffin-embedded sections of 4 µm were stained with Alcian blue and periodic acid-Schiff (PAS) and counterstained with Schiff reagent and Nuclear Fast Red solution (bar 100 µm); **b)** Representative histological images demonstrating SI structure differences between groups. Small intestine paraffin-embedded sections of 4 µm were stained with Alcian blue and periodic acid-Schiff (PAS) and counterstained with Schiff reagent and Nuclear Fast Red solution (bar 100 µm). c) Measurements of various histological parameters in the colon; d) Measurements of various histological parameters in the SI. Statistical analyses were performed using one-way ANOVA and Tukey's multiple comparison test. No statistically significant differences between groups were detected.

Methods. COL and SI samples were collected for histology and immunofluorescence analysis. For histological analysis, half of the samples were stored in Methacarn solution (60 % MeOH: 30 % Chloroform: 10 % acetic acid) for 2 hours at room temperature and dehydrated with 70 % ethanol for 2 h, prior paraffin embedding. Samples for immunohistochemistry were stored in 10 % formalin for 24 h and then dehydrated in 70 % ethanol 24 h prior the embedding process. Paraffin embedding was performed using the histokinette in a 21 h overnight cycle and was blocking was carried on using the console system TissueTek. Paraffin sections of 4 µm were done using the Leica RM2135 rotary Microtome. The analysis of goblet cells crypt depth and villi length was performed as previously described (Las Heras et al., 2019) with some modifications. Paraffin sections of 4 µm were stained with alcian blue and periodic acid-Schiff (PAS) and counterstained with Schiff reagent and Nuclear Fast Red solution. Sections were mounted in DPX mounting reagent (Sigma) and imaged using the Olympus BX51 microscope (Olympus DP71 camera), with a × 40 objective. Image analysis was performed using ImageJ. The sections were blindly scored using a light microscope (Olympus BX51, Olympus, Germany).

Supplementary material 6 –
Genes and primer sets for transcriptomic analysis in gnotobiotic mice

Supplementary Table S4. Transcriptome analysis gene targets and primer sets

Primers for host transcriptome analysis. Designed in the Assay Design Center from Roche Universal ProbeLibrary (version 2.52, 2016).

Gene Symbol	Official full name	Gene ID	mRNA Accession	Forward	Reverse	Probe
Actb	Actin, beta	11461	NM_007393	aaggccaaccgtgaaaagat	gtggtacgaccagaggcatac	#56
Lgr5	leucine rich repeat containing G protein coupled receptor 5	14160	NM_010195.2	gactttaactggagcaaagatct ca	cgagtaggttgtaagacaaatct agc	#60
Sox9	SRY (Sex determining region Y)-box 9	20682	NM_011448.4	gtacccgcatctgcacaac	ctcctccacgaagggtctct	#66
Ascl2	Achaete-scute family bHLH transcription factor 2	17173	NM_008554.3	gagagctaagcccgatgga	aggtccaccaggagtcacc	#17
Notch2	Notch 2	18129	NM_010928.2	ccatttcaagtgttcgtgtcc	cacattcatcgatgttctcttca	#40
Hes1	Hes family bHLH transcription facto 1	15205	NM_008235.2	tgccagctgatataatggagaa	ccatgataggctttgatgacttt	#20
Prmd1	PR domain containing 1, with ZNF domain	12142	NM_007548.4	acgtgtgggtacgaccttg	ccatgtccattttcatgatcc	#53
Mki67	Antigen identified by monoclonal antibody Ki 67	17345	NM_001081117.2	agggtaactcgtggaaccaa	tcttaacttcttggtgcatacaat g	#88
Vim	Vimentin	22352	NM_011701.4	ccaaccttttcttccctgaac	ttgagtgggtgtcaaccaga	#109
Egfr	Epidermal growth factor receptor	13649	NM_007912.4	gccacgccaactgtacctat	gccacacttcacatccttga	#107
Klf4	Kruppel-like factor 4	16600	NM_010637.3	cgggaagggagaagacact	gagttcctcacgccaacg	#62
Mmp2	Matrix metallopeptidase 2	17390	NM_008610.3	gtgggacaagaaccagatcac	gcatcatccacggtttcag	#85
Mmp9	Matriz metallopeptidase 9	17395	NM_013599.4	agacgacatagacggcatcc	tcggctgtggttcagttgt	#19
Ocln	Occludin	18260	NM_008756.2	gtccgtgaggcctttga	ggtgcataatgattgggtttg	#10
Tjp1	Tight junction protein 1	21872	NM_009386.2	tgcagacccagcaaaggt	ggttttgtctcatcatttcttcag	#12
Tjp2	Tight Junction protein 2	21873	NM_001198985.1	catcagcgcacacagagc	gtccctggacacaaagtcg	#1
Muc2	Mucin 2	17831	NM_023566.3	acctccaggttcaacaccag	gttggccctgttgtggtct	#10
Alpi	Alkaline phosphatase, intestinal	24197	NM_001081082.2	aaacgtggctgtcaaagcat	tcaaagaggcccatgaggt	#3
Vil1	Villin 1	22349	NM_009509.2	gatctccctgagggtgtgg	agtgaagtcttcggtggacag	#3
Chga	Chromogranin A	12652	NM_007693.2	cgatccagaaagatgatggtc	cggaagcctctgtctttcc	#58
Cdkn1a	Cyclin-dependent kinase inhibitor 1A (P21)	12575	NM_007669.5	tccacagcgatatccagaca	ggacatcaccaggattggac	#21
Reg3g	Regenerating islet-derived 3 gamma	19695	NM_011260.2	accatcaccatcatgtcctg	ggcatctttcttggcaactt	#108
Il10	Interleukin 10	16153	NM_010548	cagagccacatgctcctaga	tgtccagctggtcctttgtt	#41
Il17a	Interleukin 17A	16171	NM_010552.3	cagggagagcttcatctgtgt	gctgagctttgagggatgat	#74
Ifnγ	Interferon gamma	15978	NM_008337	atctggaggaactggcaaaa	ttcaagacttcaaagagtctgag g	#21
Fgf15	Fibroblast growth factor 15	14170	NM_008003.2	ggcaagatatacgggctgat	tccatttcctccctgaaggt	#69
Nr1h4	Nuclear receptor subfamily 1, group H, member 4	20186	NM_009108.2	gaaaatccaattcagattagtctt cac	ccgcgtgttctgttagcat	#83
Gpbar1	G protein-couple bile acid receptor 1	227289	NM_174985.1	gctagggctctcacctgga	ccccaacacagcaagaagag	#51
Cyp7a1	Cytochrome P450, family 7, subfamily a, polypeptide 1	13122	NM_007824.2	ggagcttatttcaaatgatcagg	ttggccagcactctgtaatg	#110
Abcg5	ATP binding cassette subfamily G member 5	27409	NM_031884.2	tcctgcatgtgtcctacagc	atttgcctgtcccacttctg	#31
Abcg8	ATP binding cassette subfamily G member 8	67470	NM_026180.3	aaccctgcggacttctacg	ctgcaagagactgtgccttct	#10

Supplementary material 7 –
Analysis of Colon organoids controls for viability, proliferation, apoptosis and measures of organoid size

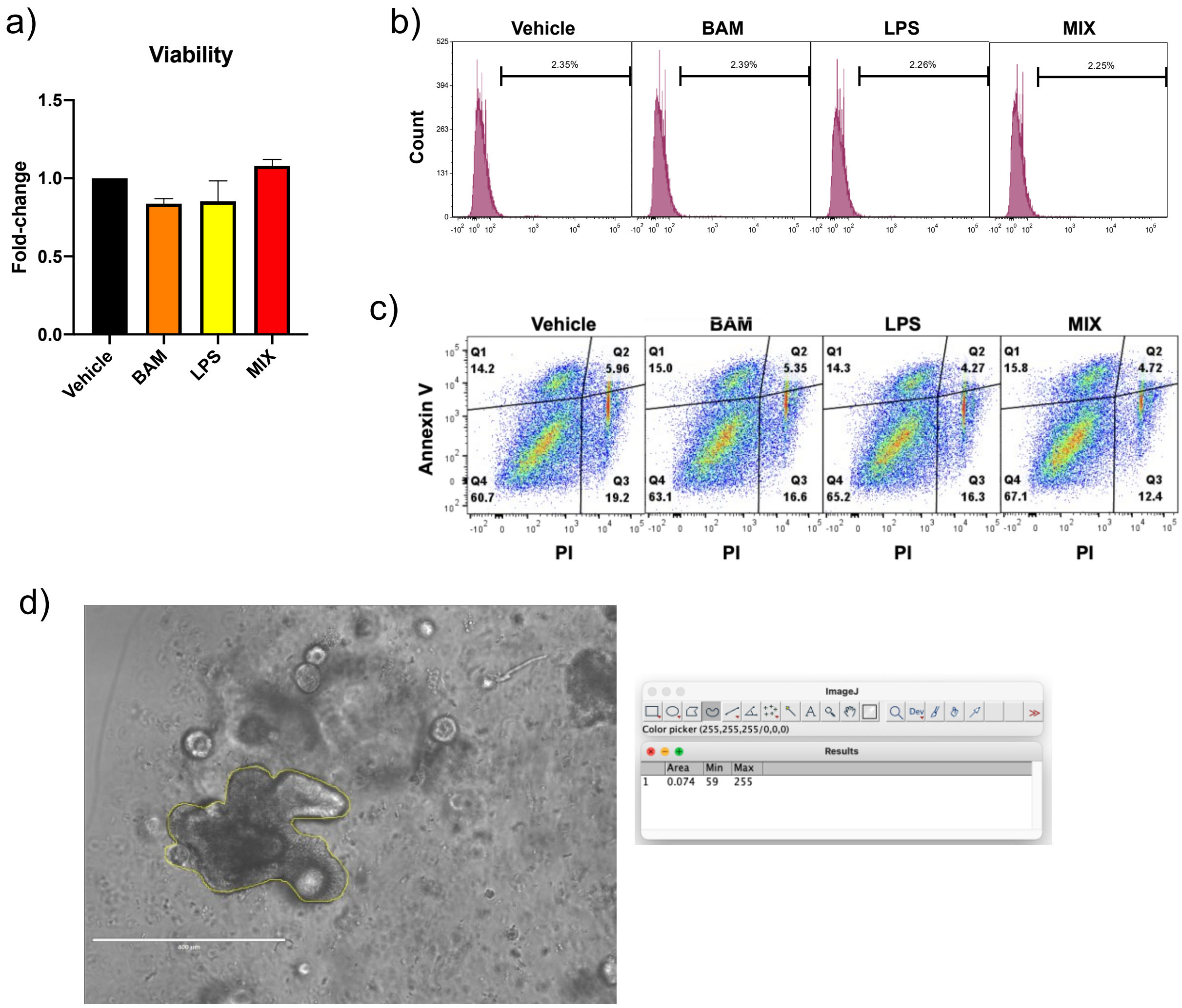

Figure Legend. a) Viability test of COLOrg exposed to different treatments. Values are represented as the fold-change between the treatments and the vehicle; b) Proliferation difference between the treatments after 7 days incubation; c) Difference in apoptosis by Annexin V/PI represented as the percentage of death cells; d) Representative image demonstrating organoid size measurements. Images from ColORG were taken by live transmission light microscopy imaging using the EVOS FL inverted microscope (Invitrogen) at 10X and 20X magnification. Acquired digital images were processed in ImageJ. ColORG were counted and surface area was calculated by outlining manually the area of ColORG. Values from each well were averaged and graphed in Figure 3.

Methods:

Crypt isolation, organoid development & treatment. Once crypts were isolated they were resuspended in the pertinent amount of Cultrex Basement Membrane Extract (BME) (R&D Systems). Twenty microliters of BME containing the crypts were seeded in 48-well plates for RT-qPCR analyses and 8 μL in 96-well plates for viability and microscopy analysis. Plates were incubated inverted at 37ºC for 30 min to allow the matrix to solidify and then conditioned L-WRN media (see Supplementary Methods) was added containing L-WRN media:Advanced DMEM/F12 1:1 supplemented with 1X Glutamax, 1% Penicillin/Streptomycin, 10 mM HEPES, 1X N2 supplement, 1X B27 supplement, 1mM N-Acetylcysteine, 50 ng/mL recombinant murine EGF, 10 mM nicotinamide, 10 mM Y-27632, 500 nM A-83-01, 10 μM SB202190 and 5 μM CHIR-99021. After 24 h media was change into mCOL media containing L-WRN media:Advanced DMEM/F12 1:1 supplemented with 1X Glutamax, 1% Penicillin/Streptomycin, 10 mM HEPES, 1X N2 supplement, 1X B27 supplement, 1mM N-Acetylcysteine, 50 ng/mL recombinant murine EGF and 10 μM Y-27632 to allow crypts for differentiation and treated with 10 mM bile acid mix (BAM; 8 μM CA, 1,5 μM UDCA, 0,5 μM CDCA), 100 ng/mL lipopolysaccharide (LPS) or LPS+BAM (MIX). Control wells with the same volume of DMSO were included. COL organoids were incubated for 7 days with changes of media every second day.After 7 days of incubation, organoid viability was evaluated by measuring the reduction of measured MTT [3-(4,5- dimethylthiazol-2-yl)-2,5-diphenyltetrazolium bromide] to formazan as described by Grabinger et al (2014) with some modifications. Cell proliferation was evaluated by flow cytometry using the Click-iT EdU Proliferation Kit (Invitrogen) as specified by the manufacturer. Apoptosis was evaluated by flow cytometry using the Annexin V/PI assay using APC-Annexin V and PI from Biolegend following manufacturer recommendations.

Reference

Grabinger T, Luks L, Kostadinova F, Zimberlin C, Medema JP, Leist M, et al. Ex vivo culture of intestinal crypt organoids as a model system for assessing cell death induction in intestinal epithelial cells and enteropathy. *Cell Death Dis* 2014; 5:e1228.

Supplementary material 8 –
Summary of findings – BSH alters gene expression profiles in the infant colon & reduces stem cell proliferation in colon organoids

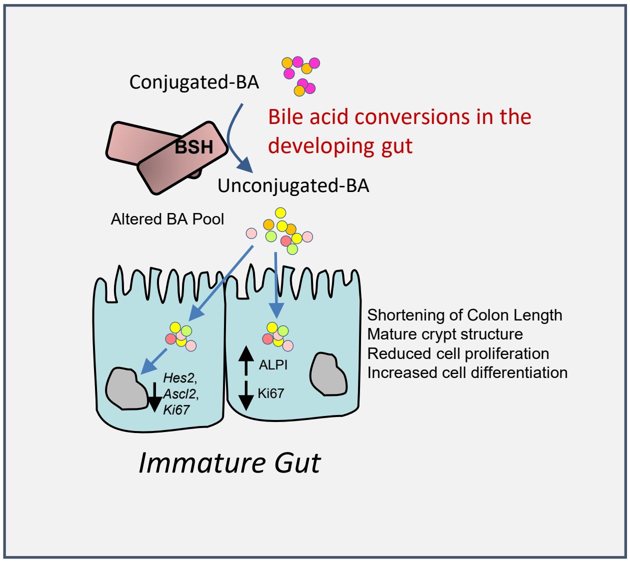

Supplement: Supplemental Material [file KGMI_A_2149023_SM9587.zip › Núñez-Sánchez Supp Material R1 (3).pdf]
