# Supplementary figures and images for: Microbial bile salt hydrolase activity influences gene expression profiles and gastrointestinal maturation in infant mice
Source: Gut Microbes. 2022 Nov 24;14(1):2149023. doi: 10.1080/19490976.2022.2149023 (PMC9704388; doi:10.1080/19490976.2022.2149023)

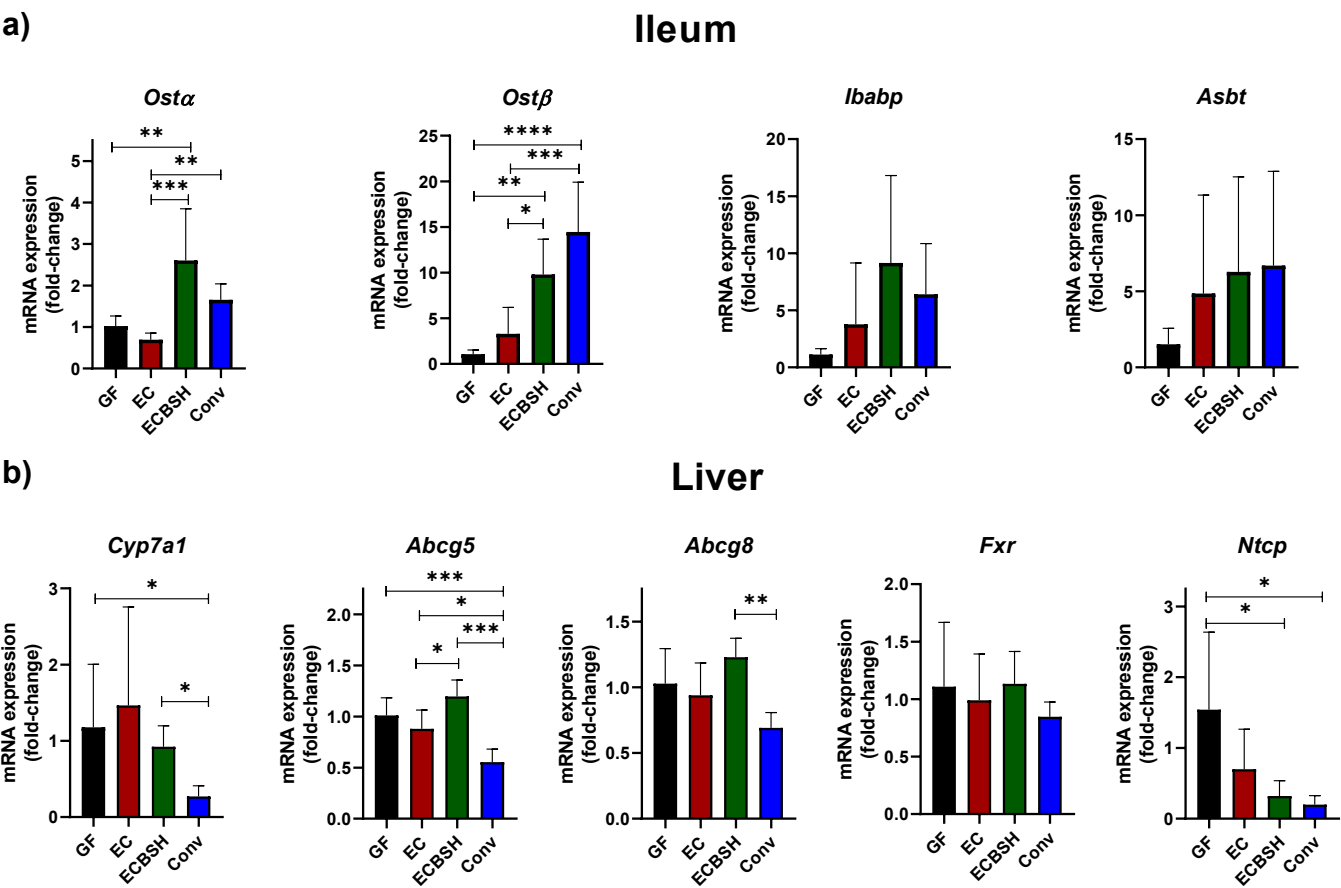

Supplement: Supplemental Material [file KGMI_A_2149023_SM9587.zip › Núñez-Sánchez Supp Material 4.pdf]
